# The cholesterol metabolite 27 hydroxycholesterol facilitates breast cancer metastasis through its actions on immune cells

**DOI:** 10.1038/s41467-017-00910-z

**Published:** 2017-10-11

**Authors:** Amy E. Baek, Yen-Rei A. Yu, Sisi He, Suzanne E. Wardell, Ching-Yi Chang, Sanghoon Kwon, Ruchita V. Pillai, Hannah B. McDowell, J. Will Thompson, Laura G. Dubois, Patrick M. Sullivan, Jongsook K. Kemper, Michael D. Gunn, Donald P. McDonnell, Erik R. Nelson

**Affiliations:** 1 Department of Molecular and Integrative Physiology, University of Illinois at Urbana-Champaign, 407S Goodwin Avenue (MC-114), Urbana, IL 61801 USA; 2 Department of Medicine, Division of Cardiology, Duke University School of Medicine, 346 Sands Building, Durham, NC 27710 USA; 3 Department of Pharmacology and Cancer Biology, Duke University School of Medicine, Box 3813, Durham, NC 27710 USA; 4 Proteomics and Metabolomics Shared Resources, Duke University School of Medicine, B02 Levine Science Research Center, 450 Science Drive, Durham, NC 27708 USA; 5 Department of Medicine, Duke University School of Medicine, Durham, 27710 NC USA; 6 Geriatric Research, Education and Clinical Center, Durham Veterans Affairs Medical Center, 508 Fulton Street, Durham, NC 27705 USA; 7 Department of Medicine, Division of Cardiology, Duke University School of Medicine, 346 Sands Building, Durham, NC 27710 USA; 8 University of Illinois Cancer Center, Chicago, IL 60612 USA; 9 Division of Nutritional Sciences, University of Illinois at Urbana-Champaign, Urbana, IL 61801 USA

## Abstract

Obesity and elevated circulating cholesterol are risk factors for breast cancer recurrence, while the use of statins, cholesterol biosynthesis inhibitors widely used for treating hypercholesterolemia, is associated with improved disease-free survival. Here, we show that cholesterol mediates the metastatic effects of a high-fat diet via its oxysterol metabolite, 27-hydroxycholesterol. Ablation or inhibition of CYP27A1, the enzyme responsible for the rate-limiting step in 27-hydroxycholesterol biosynthesis, significantly reduces metastasis in relevant animal models of cancer. The robust effects of 27-hydroxycholesterol on metastasis requires myeloid immune cell function, and it was found that this oxysterol increases the number of polymorphonuclear-neutrophils and γδ-T cells at distal metastatic sites. The pro-metastatic actions of 27-hydroxycholesterol requires both polymorphonuclear-neutrophils and γδ-T cells, and 27-hydroxycholesterol treatment results in a decreased number of cytotoxic CD8^+^T lymphocytes. Therefore, through its actions on γδ-T cells and polymorphonuclear-neutrophils, 27-hydroxycholesterol functions as a biochemical mediator of the metastatic effects of hypercholesterolemia.

## Abstract (web summary)

High cholesterol is a risk factor for breast cancer recurrence. Here the authors show that cholesterol promotes breast cancer metastasis via its metabolite 27-hydroxycholesterol (27HC) that acts on immune myeloid cells residing at the distal metastatic sites, thus promoting an immune suppressive environment.

## Introduction

Obesity is an established risk factor for the onset of breast cancer, and in patients with established disease, it is associated with a decreased time to recurrence and poorer overall survival^1, 2^. The significance of the association between obesity and metastatic recurrence is highlighted by the fact that >90% of breast cancer mortality is attributable to metastasis. However, the multifactorial nature of obesity has made it difficult to establish cause and effect relationships with respect to breast cancer pathophysiology. Proposed mechanisms include obesity-associated increases in circulating levels of insulin, insulin like growth factor 1 or inflammatory cytokines/adipokines released from adipose-infiltrating immune cells or adipose itself^3^. For estrogen receptor alpha (ERα)-positive breast cancer, the local production of estrogens (17-β estradiol or estrone) by aromatase expressed in adipose tissue is likely to be a contributing factor. Elevated cholesterol is a comorbidity of obesity^4–6^, generating the postulate that cholesterol may mediate some of the pro-metastatic effects of obesity. Epidemiologic data regarding cholesterol and breast cancer onset are controversial, and it is not clear whether total, LDL or HDL cholesterol impart risk^7–9^. Studies investigating the correlation between patients taking inhibitors of 3-hydroxy-3-methylglutaryl coenzyme A reductase, statins and risk of onset are equally conflicting, with the largest meta-analyses indicating that there is no association^10^. However, there is strong clinical evidence supporting a role for cholesterol in breast cancer recurrence and survival. Elevated total cholesterol is associated with increased breast cancer recurrence^11^. Further, several retrospective studies indicate patients taking statins, demonstrate a significantly increased time to breast cancer recurrence^12–14^. Finally, in a recently published phase III, double-blind trial including 8010 postmenopausal women with early-stage, hormone receptor-positive invasive breast cancer, it was found that taking cholesterol lowering medication during endocrine therapy was associated with increased recurrence-free survival time and distant recurrence–free interval^15^. Considering these observations, we hypothesized that cholesterol is a mediator of some of the effects of obesity on breast cancer metastasis.

Previously we have shown that a high-cholesterol diet can increase the growth of ER-positive tumors in the murine MMTV-PyMT model, and that statin treatment could attenuate the effects of a high-fat diet on E0771 tumor growth^16^. Notable was the observation that the primary metabolite of cholesterol, 27-hydroxycholesterol (27HC), behaved as a selective estrogen receptor modulator (SERM) that exhibited agonist activity in breast cancer cells and as such was able to promote the growth of ER-positive tumors^16, 17^. Importantly, 27HC levels have been found to be elevated within breast tumors compared to normal breast tissue, increased protein expression of the enzyme responsible for its synthesis (CYP27A1) is associated with a higher tumor grade, and circulating 27HC levels were elevated in patients treated with an aromatase inhibitor^16–19^. In addition to its effects on primary tumor growth, elevated 27HC also increased metastatic burden. Somewhat unexpectedly, the pro-metastatic effects of 27HC did not appear to involve ER, while activation of the liver X receptors (LXRs) was implicated. Indeed, it was demonstrated that synthetic LXR agonists could also induce breast cancer cell metastasis albeit less effectively than 27HC. Thus, it appeared likely that in addition to LXR activation, 27HC engaged additional targets that contributed to the metastatic phenotype. Therefore, the goals of this study were to (1) determine if cholesterol increases metastasis independent of a high-fat diet, (2) establish the role of 27HC as a biochemical mediator of cholesterol associated metastasis, and (3) elucidate the mechanisms by which 27HC increases metastasis.

Herein, we report that an isocaloric diet high in cholesterol (HCD) alone was sufficient to increase metastasis in several pre-clinical models of mammary cancer, firmly establishing a causative effect of cholesterol on metastasis. This activity could be attributed to effects of the cholesterol metabolite 27HC on myeloid cell function and was associated with increased numbers of polymorphonuclear neutrophils (PMNs) and γδ T cells, and decreased cytotoxic CD8^+^ T cells within tumors and metastatic lesions. Importantly, these studies also highlight the potential clinical utility of interfering with the production and/or activity of cholesterol and 27HC in patients with breast cancer and possibly other solid tumors.

## Results

### 27HC mediates obesity and cholesterol increase in metastasis

**Fig. 1:**
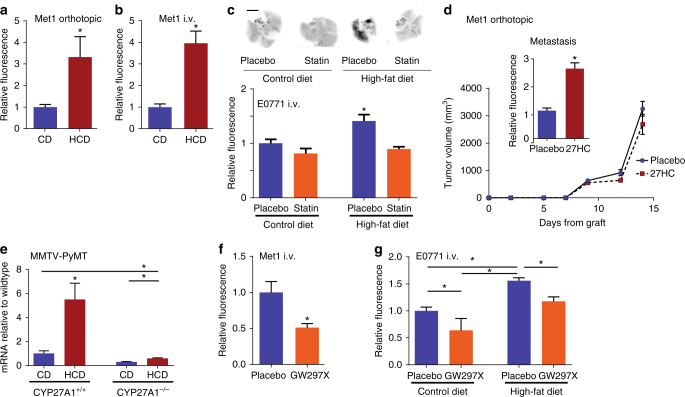
27HC is a biochemical mediator of the effects of obesity and elevated cholesterol on breast cancer metastasis. **a** A high-cholesterol diet (HCD) increases spontaneous metastasis of murine ER-negative Met1 cells (*N* = 10 each). **b** Mice fed a high-cholesterol diet (HCD) exhibit increased lung colonization of i.v. grafted Met1 mammary tumors (CD *N* = 7, HCD *N* = 8). **c** A high-fat diet (HFD) increases lung colonization of murine E0771 i.v. grafts in *APOE3* mice (i.v. graft of cells). Pretreatment for 7 days prior to the graft and continued treatment throughout the study with daily placebo or atorvastatin (statin, 40 mg/kg) by oral gavage reduced metastatic colonization. Representative images of iRFP-expressing E0771 colonies (depicted as *black*) within the lungs are presented above quantified data (control diet (CD) placebo *N* = 11, statin *N* = 7, HFD placebo *N* = 8, statin *N* = 7, *scale bar* indicates 5 mm). **d** Chronic administration of exogenous 27-hydroxycholesterol (27HC) increases spontaneous metastasis to the lung as measured by relative fluorescence (*inset*) without impacting growth rate of the primary tumor (*N* = 3). **e** Metastatic effects of a HCD in MMTV-PyMT mice requires conversion to 27HC by CYP27A1 (wild-type CD *N* = 8, HCD *N* = 14, CYP27A1^−/−^ CD *N* = 3, HCD *N* = 9). Quantitative RT-PCR for PyMT transcript within the lung is presented in the graph and used to quantify metastasis since its expression should be restricted to mammary epithelial cells. **f** Chronic treatment with a small molecule inhibitor of CYP27A1, GW273297X (GW297X) decreases colonization of lung tissue from Met1 i.v. grafts (*N* = 4 each). **g** Lung colonization from E0771 i.v. grafts in *APOE3* mice is increased by a HFD, but attenuated in mice chronically treated with GW297X (CD placebo *N* = 5, GW297X *N* = 3, HFD placebo *N* = 7, GW297X *N* = 6). Results are depicted as mean +/− SEM. *Lines* and *asterisks* denote statistical differences between groups (*p* < 0.05). [**a**, **b**, **d**, **f**: Unpaired two-tailed student’s *t*-test. **c**, **e**, **g**: One-way ANOVA followed by a Student Newman-Keuls multiple comparison test]

There is compelling evidence that high-fat diets (HFD) increase tumor growth and metastasis in established models of breast cancer^20, 21^. We have previously demonstrated that inhibiting cholesterol biosynthesis was sufficient to mitigate the impact of a HFD on tumor growth. Leveraging these results we have explored the relationship between a HFD, elevated cholesterol, and metastasis, using relevant in vivo models of breast cancer. We demonstrated that a high-cholesterol diet alone was sufficient to increase spontaneous lung metastasis from orthotopically engrafted Met1 tumors (ER-negative breast cancer cells), without a significant impact on the growth of the primary tumor (Fig. 1a; Supplementary Fig. 1). Similarly, lung colonization by Met1 cells grafted intravenously (i.v.) was also increased in mice on a high-cholesterol diet (Fig. 1b). The impact of inhibiting cholesterol biosynthesis on the ability of a HFD to increase breast cancer metastasis was next assessed. For these studies, we made use of a transgenic model in which the murine *Apoe* gene was replaced with the human *APOE3* allele (APOE3 mice)^22^, as wild-type mice do not exhibit hypercholesterolemia when fed a HFD. Using this approach we have previously demonstrated that a HFD significantly elevates circulating cholesterol and that this can be inhibited by statin administration^16^. Importantly, a HFD resulted in increased metastatic burden when these mice were grafted with syngeneic E0771 cells, a murine model of ER-positive mammary cancer (Fig. 1c, Supplementary Fig. 2). Oral treatment with atorvastatin completely attenuated the effect of a HFD on metastasis, thus implicating cholesterol as a causative agent in this process. Altogether these data implicate cholesterol as a biochemical link between HFD and increased metastasis.

Previously, we and others have shown that the ability of cholesterol to promote breast tumor growth required its conversion by the enzyme CYP27A1 to the oxysterol 27-hydroxycholesterol (27HC)^16, 17^. This effect of 27HC was shown to result from its ability to function as a partial agonist of ERα.

It was thus important to determine whether the effect of hypercholesterolemia on metastasis was also dependent on 27HC, and if this activity required ERα. To this end, the effects of 27HC on the metastasis of the ER-negative cell line, Met1, was examined. Notably, absent an effect on the growth rate of the primary tumors, 27HC increased the spontaneous metastasis from this orthotopic tumor model (Fig. 1d). Furthermore, the pro-metastatic effects of a HCD in MMTV-PyMT mice required the presence of CYP27A1 (Fig. 1e; Supplementary Fig. 3). Even when on a normal chow (low cholesterol) diet, lung metastasis was considerably reduced in PyMT^+^;CYP27A1^−/−^ mice when compared to their wild-type counterparts. The importance of 27HC in this latter activity was confirmed by demonstrating that treatment with GW273297X, a small molecule inhibitor of CYP27A1, decreased the spontaneous metastasis of Met1 tumors to the lung, in normocholesterolemic mice on normal chow (Fig. 1f). Similarly, GW273297X attenuated the effects of a HFD on lung-colonization from E0771 grafts in the APOE3 mouse model (Fig. 1g; Supplementary Fig. 2). The efficacy of GW273297X in inhibiting 27HC synthesis has been previously documented in vitro and in vivo, and was confirmed here (Supplementary Fig. 4)^16, 23^. While GW273297X significantly reduced circulating 27HC levels in HFD APOE3 mice, diet and treatments also led to subtle changes in 24R/S-HC and 22S-HC. It is possible that GW273297X may have led to alterations in other cholesterol metabolite levels including those with anti-tumor properties such as the dendrogenin A, and that these may be responsible for its activity^24^. It should be noted that the oxysterol content of the diets was not specifically determined, and that accumulation of different oxysterols within the diet could also contribute to the observed phenotype. Regardless, our data, when considered in totality, supports a role for 27HC as a downstream mediator of the pro-metastatic effects of a HFD and cholesterol, and this activity is independent of the expression of ERα within cancer cells. These findings, confirmed in multiple models of mammary cancer, provide strong rationale for the development of CYP27A1 inhibitors for use in the prevention and/or treatment of metastatic breast cancer.

### 27HC promotes metastasis in a tumor cell extrinsic manner

**Fig. 2:**
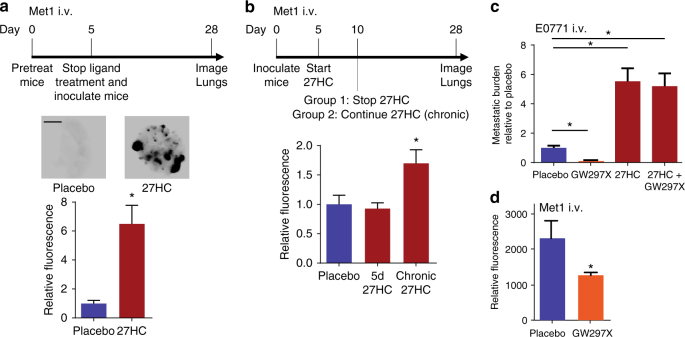
Effects of 27HC are mediated by its cancer-cell extrinsic effects on the host. **a** Mice were pretreated with placebo or 27HC (20 mg/kg) daily for 5 days, at which point Met1 cells expressing iRFP were grafted (i.v.) and treatment ceased. At day 28, lungs were harvested, imaged and iRFP fluorescence quantified. Experimental timeline outlined above. Representative images of iRFP-expressing Met1 colonies (depicted as *black*) within the lungs are presented above quantified data (placebo *N* = 5, 27HC *N* = 4, *scale bar* indicates 5 mm). **b** Mice were grafted (i.v.) with Met1 cells expressing iRFP on day 0. On day 5, mice were treated daily with placebo or 27HC for either 5 days or for the remainder of the study (chronic 27HC). At day 28, lungs were harvested, imaged and iRFP fluorescence quantified. Experimental timeline outlined above quantified data (placebo *N* = 7, 5 days 27HC *N* = 7, chronic 27HC *N* = 6). **c** Pretreatment of naive mice (regime outlined in **a**) with the CYP27A1 inhibitor GW297X (100 mg/kg) reduces colonization of lung by E0771 cells, which is reversed by co-pretreatment with 27HC (*N* = 5/group). **d** Pretreatment of mice with GW297X (as in **c**) results in decreased Met1 colonies. For this experiment, mice were pretreated with placebo or GW297X (100 mg/kg) for 5 days at which point Met1 cells expressing iRFP were grafted (i.v.) and treatment ceased. Five days post-graft, lungs were harvested, imaged, and fluorescence quantified (placebo *N* = 9, GW297X *N* = 10). Results are depicted as mean +/− SEM. *Lines* and *asterisks* denote statistical differences between groups (*p* < 0.05). **a**, **d**: Unpaired two-tailed student’s *t*-test. **b**, **c**: One-way ANOVA followed by a Student Newman-Keuls multiple comparison test

The effects of 27HC on primary tumor growth require ERα and 27HC-stimulated growth was inhibited with specific antiestrogens, indicating that this activity occurs in a tumor cell intrinsic manner^16, 17^. However, the dramatic effect of 27HC on the metastasis of both ERα-positive and ERα-negative tumors suggests that an alternative target is also engaged, raising the question as to whether these activities are manifest in a tumor cell intrinsic manner or if host targets are also involved. In general support of a tumor cell extrinsic activity for 27HC/HCD was the observation that a HCD increased the colonization of lung tissue by ER-negative cancer cells when injected intravenously (i.v.), an approach that would be expected to minimize the cancer cell intrinsic effects of cholesterol (Fig. 1b). To address this possibility in a more definitive manner, naive wild-type mice were pretreated with either placebo or 27HC, followed by the injection of Met1 mammary cancer cells (i.v.) at such time 27HC treatment was ceased. The results of this experiment shown in Fig. 2a indicated that the 27HC pretreated mice had significantly more metastatic nodules than those that were pretreated with placebo (*p* = 0.016, Mann–Whitney test). In this pretreatment model, plasma levels of 27HC reach 0.334 µM, which is well within circulating levels found in humans^25^, and decrease after cessation of injections (Supplementary Fig. 5). These findings are consistent with 27HC having its primary effects on metastasis through its actions in cells other than the cancer cells themselves. This hypothesis was tested in an additional experiment in which Met1 cells were engrafted (i.v.) into treatment-naive mice and treatment was initiated 5 days post-graft, with either placebo or 27HC, allowing for colonization prior to ligand administration. After 5 days 27HC treatment was discontinued in one group but continued in another cohort. No increase in metastatic burden was observed in mice treated with 27HC for 5d post-graft, indicating that the previously observed effects of 27HC were indeed due to its ability to ‘‘prime’’ the distal metastatic site (Fig. 2b). Interestingly, however, chronic treatment with 27HC starting post-graft resulted in a small but significant (*p* < 0.01, Student Newman-Keuls (SNK) test) increase in metastatic burden, potentially due to increased secondary metastatic seeding. At this point it is not known why this increase is smaller than that observed for pretreated mice, but may be due to the increased concentration of circulating cancer cells in the pretreatment model. Basal concentrations of 27HC were found to be important in priming the metastatic microenvironment, as pretreatment with the CYP27A1 inhibitor GW273297X also reduced metastasis as determined by visual assessment and confirmed by messenger RNA (mRNA) analysis of genes enriched in epithelial cancer cells (Fig. 2c and Supplementary Fig. 6). The effects of GW273297X were reversed by co-treatment with exogenous 27HC confirming that the anti-colonization effects of this drug were mediated through its inhibition of CYP27A1. Interestingly, the anti-colonization effects of CYP27A1 inhibition were apparent as early as 5 days after cell engraftment (Fig. 2d, Supplementary Fig. 7). We, therefore, conclude that the pro-metastatic effects of 27HC are likely manifest in a tumor cell extrinsic manner.

### Myeloid cells required for the metastatic effects of 27HC

**Fig. 3:**
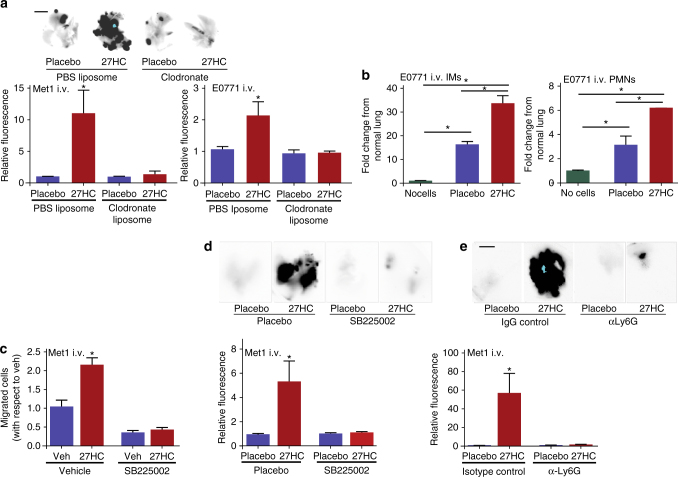
Metastatic effects of 27HC require polymorphonuclear neutrophils. **a** Myeloid-derived immune cells are required for the colonizing effects of 27HC, as clodronate-liposome depletion of phagocytic cells ablates its effects. Mice were pretreated prior to cellular graft (i.v.) with placebo or 27HC and/or liposomes containing either PBS or clodronate as outlined in Fig. 2a. Representative images of iRFP-expressing Met1 colonies (depicted as *black*) within the lungs are presented above quantified data for both Met1 and E0771 mammary cancer models (Met1 in order from *left* to *right N* = 5, 3, 3, 4, E0771 in order from *left* to *right N* = 3, 3, 4, 4, *scale bars* indicate 5 mm). **b** 27HC treatment of metastasis bearing mice results in the recruitment of inflammatory monocytes (IMs) and polymorphonuclear neutrophils (PMNs) to the lung, as assessed by FACS analysis (in order from *left* to *right N* = 3, 3, 6). For comparison, one group of mice received only an i.v. PBS injection (no cells). **c** 27HC increases in vitro migration of bone marrow-derived neutrophils in a CXCR2-dependent manner, as the small-molecule inhibitor of CXCR2, SB225002 attenuates its effects (*N* = 3/group). **d** Colonizing effect of 27HC requires activation of CXCR2. Mice were pretreated with indicated ligands for 5 days prior to engraftment with Met1 cells expressing iRFP (as in Fig. 2a). Representative images of iRFP-expressing Met1 colonies (depicted as *black*) within the lungs are presented above quantified data (*N* = 6/group, *scale bar* indicates 5 mm). **e** PMNs are required for the colonizing effects of 27HC, as immune-depletion of PMNs with an antibody against Ly6G (α-Ly6G) ablates its effects. Mice were pretreated with indicated ligands for 5 days prior to engraftment with Met1 cells expressing iRFP (as in Fig. 2a). Representative images of iRFP-expressing Met1 colonies (depicted as *black*) within the lungs are presented above quantified data (placebo groups *N* = 4, isotype control 27HC group *N* = 6, α-Ly6G 27HC group *N* = 7, *scale bar* indicates 5 mm). Results are depicted as mean +/− SEM. *Lines* and *asterisks* denote statistical differences between groups (*p* < 0.05). [One-way ANOVA followed by a Student Newman-Keuls multiple comparison test]

Myeloid derived cells are key regulators of the metastatic process^26^, not only in the primary tumor, but also at the distal metastatic site, where they facilitate the establishment and subsequent growth of metastatic lesions^27, 28^. Since 27HC is a circulating metabolite with the capacity to exert effects at the distal metastatic site, and recent evidence shows that steroid hormones can alter myeloid cell populations within the tumor microenvironment^29^, we speculated that the metastatic phenotype observed in 27HC pretreated mice may involve cells of the myeloid lineage. In order to test this hypothesis, we repeated the pretreatment experiment outlined in Fig. 2a, but this time in the presence or absence of a strategy that would acutely deplete phagocytic cells of the myeloid lineage. Specifically, 24 and 4 h before cell graft, mice were treated with liposomes containing either phosphate-buffered saline (PBS) or clodronate; clodronate liposomes being preferentially taken up by phagocytic cells, thereby eliminating them (Supplementary Fig. 8). As clearly demonstrated in Fig. 3a, the colonizing effect of 27HC was completely attenuated in mice lacking myeloid cells. This effect was consistent between two murine mammary cancer models: Met1 and E0771.

Tumor-associated macrophages are a collection of functionally distinct myeloid cells within the tumor, which drive tumor progression and metastasis^30–32^. Indeed, we have previously shown that the myeloid cell repertoire differs among different tumor types and this is established in large part by the associated tumor and host-specific factors^32^. Of specific importance to this study was our previous observation in E0771 tumors that the composition of myeloid cell types recruited to the distal metastatic sites were similar to that of the primary tumor^32^, with the exception of inflammatory monocytes (IMs), which were relatively more abundant in the primary tumor. A comprehensive analysis of the leukocyte cell composition within the lungs of placebo and 27HC-treated mice bearing metastatic lesions showed that many leukocyte types changed in relative concentration between metastasis bearing lungs and those free of metastasis (Supplementary Fig. 9). However, among mice bearing lung metastases, 27HC treatment consistently resulted in a robust increase in the numbers of CD11b^high^; Ly6C^+^; CCR2^+^ “inflammatory monocytes” (IMs), and Ly6G^+^ PMNs (Fig. 3b). Consistent with this finding, treatment with the CYP27A1 inhibitor decreased the presence of these populations (Supplementary Fig. 7). In subsequent analyses we have defined IMs as CD11b^high^; Ly6C^high^; Ly6G^low-negative^.

We have previously demonstrated that metastatic lungs exhibit a dramatic increase in PMNs compared to IMs, implicating PMNs in the pathogenesis of metastasis, and indicating that IMs may be ‘‘responding’’ cells rather than initiating cells^32^. Furthermore, recent studies have demonstrated critical roles for PMNs in breast cancer metastasis formation^33, 34^. Therefore, we tested whether the effects of 27HC were mediated by PMNs. It has been demonstrated by others that PMN cell trafficking to sites of inflammation is regulated in part by processes downstream of the chemokine receptor CXCR2^35^. Furthermore, it has been reported that oxysterols can interact with and activate CXCR2^36^. Thus, we first investigated whether 27HC had the capacity to increase the migration of primary PMNs in an in vitro model. As shown in Fig. 3c, 27HC increased the migration of primary bone-derived neutrophils, and this activity was quantitatively inhibited by co-treatment with SB225002, a small molecule CXCR2 antagonist. The involvement of CXCR2 was confirmed using PMNs isolated from CXCR2^fl/fl^ and infected with lentivirus containing an expression vector for Cre-recombinase (Supplementary Fig. 10). The migratory stimulation by 27HC was consistent in two cell lines representing myeloid cell precursors (RAW264.7 and U937 cells; Supplementary Fig. 10). Further, the migratory induction by 27HC was unchanged when 27HC was added to the bottom chamber or top chamber, indicating that 27HC might not be a chemoattractant per se. Importantly, 27HC-dependent metastasis of Met1 cells following tail vein injection was also inhibited by pretreatment with SB225002 (Fig. 3d).

To specifically test whether the pro-metastatic effects of 27HC required PMNs, we adopted an immune-depletion strategy to remove Ly6G^+^ cells. Mice were pretreated with placebo or 27HC for 5 days. Thirty-six and 12 h prior to cell engraftment, mice were injected with either purified anti-Ly6G antibody or a corresponding isotype control. Successful immune depletion of PMNs at the time of graft was confirmed by flow cytometry (Supplementary Fig. 11). Importantly, the pro-metastatic effects of 27HC were completely absent in mice whose PMNs were depleted, strongly supporting our hypothesis that 27HC-recruited PMNs facilitate metastasis (Fig. 3e). On the other hand, while colonization was reduced when IMs were either depleted with an antibody against Ly6C, or their recruitment was reduced with the CCR2 antagonist RS-504393, 27HC continued to stimulate colonization (Supplementary Fig. 12).

### PMNs and γδ-T cells mediate metastatic effects of 27HC

**Fig. 4:**
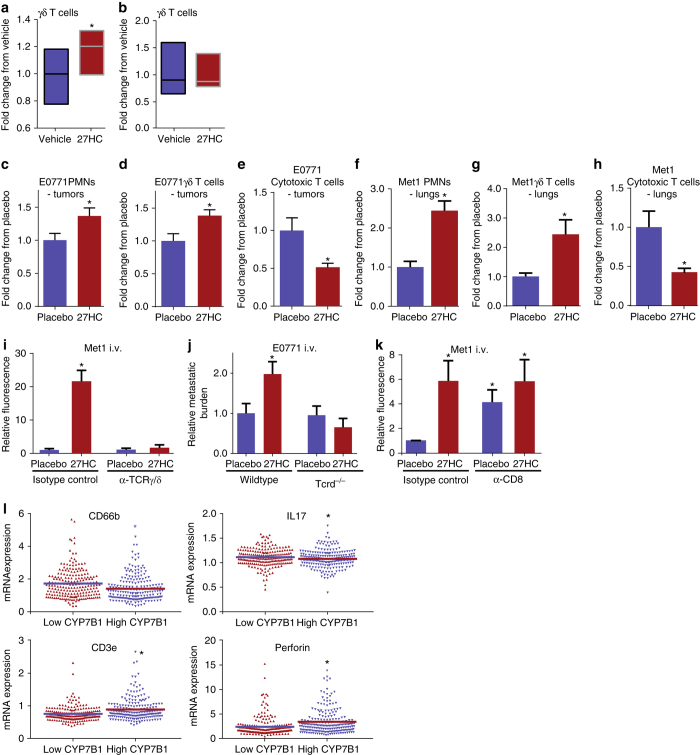
27HC increases presence of γδ-T cells and PMNs, and decreases presence of cytotoxic CD8+ T cells within tumors and metastatic lungs. **a** Bone marrow-derived neutrophils were co-cultured with splenocytes in the presence or absence of 27HC for 4 days. At this time, cells were analyzed by flow cytometry for the presence of γδ-T cells (*N* = 5/group). **b** The 27HC stimulated increase in γδ-T cells is not apparent when only splenocytes were cultured. **c**–**e** Early-stage E0771 tumors from mice treated with placebo or 27HC were analyzed by flow cytometry for γδ-T cells, PMNs and cytotoxic T cells (placebo *N* = 10, 27HC *N* = 9). **f**–**h** Early-stage metastatic lungs (Met1) from mice treated with placebo or 27HC were analyzed by flow cytometry (*N* = 8/group). **i** γδ-T cells are required for the colonizing effects of 27HC, as immune-depletion of γδ-T cells with an antibody against TCRγδ (α-TCRγ/δ) ablates its effects on Met1 colonization of the lungs (*N* = 8 except for isotype control 27HC group where *N* = 10). Mice were pretreated with indicated ligands for 5 days prior to engraftment with Met1 cells expressing iRFP (as in Fig. 2a). **j** γδ-T cells are required for the colonizing effects of 27HC, as 27HC fails to increase colonization in Tcrd^−/−^ mice (from *left* to *right N* = 13, 12, 12, 13). **k** Immune depletion of CD8^+^ cells (α-CD8) increases colonization, and exogenous 27HC treatment does not further increase colonization of Met1 cells to the lungs (from *left* to *right*: *N* = 9, 7, 8, 8). **l** Scatterplot analysis of TCGA data of 421 invasive breast cancer tumors for mRNA expression of CD66b, IL-17, CD3e, and PRF1 (perforin), parsed by median expression of CYP7B1. Results are depicted as mean +/− SEM, with the exception of **a**, **b**, and **l** where the *horizontal line* depicts the mean. *Asterisks* denote statistical differences between groups (*p* < 0.05). [**a**–**h**: Unpaired two-tailed student’s *t*-test. **i**–**k**: One-way ANOVA followed by a Student Newman-Keuls multiple comparison test. **l**: Mann–Whitney test]

It has been shown previously that by secreting interleukin-17 (IL-17), γδ-T cells can induce the expansion and polarization of granulocytes. These polarized PMNs are then enabled to actively suppress cytotoxic CD8^+^ T cells^33^. We adopted a method to enrich for PMNs from bone marrow and confirmed their viability in cell culture (Supplementary Fig. 13). Using an in vitro co-culture approach wherein bone marrow-derived cells enriched for PMNs by density gradient centrifugation were cultured with splenocytes, it was determined that 27HC treatment resulted in an increase in the numbers of rare γδ-T cells compared to vehicle treated cells, most likely due to the increased expansion of this cell type (Fig. 4a). Notably, the expansion of γδ-T cells driven by 27HC required the presence of excess neutrophils, as culturing of splenocytes alone with 27HC did not induce γδ-T-cell proliferation (Fig. 4b). This suggests that unlike previous reports^33^, neutrophils have a role in stimulating γδ-T cell growth in the presence of 27HC. However, the main effector cell of 27HC signaling has yet to be determined. Importantly, modeling colonization by analyzing early stage tumors, a critical stage for immune-suppression, it was further determined that treatment with 27HC also increased the number of both intratumoral γδ-T cells and PMNs (Fig. 4c, d). A corresponding decrease in CD8^+^ T cells was also observed (Fig. 4e), consistent with the hypothesis that 27HC does indeed suppress cytotoxic immune cell activity. Similarly, we found that 27HC increased the presence of γδ-T cells and decreased cytotoxic T cells within the lungs, 24 h post i.v. inoculation with Met1 cells (Fig. 4f–h). Immune ablation of γδ-T cells did not have a significant impact on basal colonization of Met1 cells (*p* > 0.05, SNK test), but completely attenuated 27HC-induced colonization, indicating that in addition to PMNs, these cells are critical mediators of 27HC (Fig. 4i, Supplementary Fig. 14). These effects were corroborated using the E0771 model in mice void of γδ-T cells (Tcrd^tm1/mom^/J, also known as TCRD^−/−^) (Fig. 4j). Interestingly, and in support of our co-culture assays, we found that 27HC failed to increase γδ-T cells in the absence of PMNs, while it continued to promote an increase of PMNs in the metastatic lungs of mice immune depleted of γδ-T cells (Supplementary Fig. 15). This would indicate that PMNs are the primary effector cells of 27HC and that γδ-T cells are ‘‘responding cells’’ the presence of which are required for the full effects of 27HC. Previous studies have shown that metastasis-associated PMNs have an increased expression of Prok2, NOS2, S100A8 and S100A9^33^. When bone marrow derived PMNs were cultured, 27HC modestly induced the mRNA expression of Prok2 and NOS2, while having no effect on S100A8 or S100A9 (Supplementary Fig. 16). Likewise, 27HC did not significantly regulate the expression of these genes in splenocytes (*p* > 0.05, SNK test). However, when PMNs were co-cultured with splenocytes (similar to Fig. 4a), the induction of Prok2 and NOS2 by 27HC was significantly enhanced, and S100A8 and S100A9 were now both induced (*p* < 0.05, SNK test). The enhanced induction of these genes was not apparent when splenocytes from TCRD^−/−^ mice were co-cultured with wild-type bone marrow (Supplementary Fig. 16). Collectively, this data suggests that 27HC results in increased PMNs at the metastatic site, but that γδ-T cells are required for their full pro-tumorigenic properties. However, further experimentation is now required to test this model and determine the precise interactions between PMNs and γδ-T cells.

Immune depletion of CD8^+^ T cells resulted in increased basal colonization which 27HC did not increase further (Fig. 4k). Therefore, it is likely that through its actions on PMNs and/or γδ-T cells, 27HC suppresses cytotoxic CD8^+^ cells, thereby allowing cancer cells to escape immune surveillance at distal metastatic sites. Highlighting the clinical relevance of our findings are data from the TCGA, which indicates that in patients with low tumoral CYP7B1 (and thus expected elevated 27HC) there is increased expression of the granulocyte marker CD66b and the PMN polarizing factor IL-17 (Fig. 4l). On the other hand, low CYP7B1 expression is also associated with decreased expression of CD3e and perforin, markers of T-cell infiltration and cytotoxic activity, respectively.

### 27HC increases metastasis of other solid tumor types

The results presented thus far suggest that the effects of 27HC on metastasis are due in large part to its actions on host cells. Thus, we speculated that the pro-metastatic effects of 27HC may also increase metastasis of cancer cells other than those derived from breast tumors. Using i.v. injection of cancer cells as a model of metastasis, it was determined that 27HC pretreatment increased the number of metastatic nodules in the lungs when syngeneic colorectal cancer cells (MC38), lung cancer cells (Lewis Lung), melanoma (B16-F0) or pancreatic cells (KPC915) were injected (Supplementary Fig. 17). Increased colonization of pancreatic cancer cells to the heart and liver, or melanoma cells to the liver, were also observed in 27HC-treated mice. Further, the number of nodules formed after injection of colorectal cancer cells into wild-type mice was significantly higher than in mice that lack the ability to synthesize 27HC (CYP27A1^−/−^ mice; *p* = 0.03). Considering these studies performed in well-validated models, it is concluded that cholesterol, secondary to its conversion to 27HC, is likely to impact metastasis of a broad range of tumor types; a result that highlights the importance of this axis as a therapeutic target.

## Discussion

Considerable epidemiological data links both obesity and elevated cholesterol with an increased likelihood of recurrence in breast cancer patients, while statins have been shown to significantly improve prognosis. Although several studies have indicated that ‘‘Western diets’’ high in both fat and cholesterol increase mammary cancer metastasis^8^, the specific contributions of cholesterol in this process have not been evaluated. Therefore, using clinically relevant murine models of breast cancer, we show that (1) elevated cholesterol alone can increase metastasis, (2) the metastatic effects of obesity are mediated in part by elevations in cholesterol, and (3) statin intervention can attenuate the effects of a high-fat diet. Furthermore, it was determined that the effect of dietary cholesterol on metastasis requires CYP27A1, the enzyme responsible for the synthesis of 27HC. In support of the idea that the 27HC signaling axis mediates the effects of cholesterol, it was shown that exogenous supplementation with 27HC robustly increased lung metastasis from tumors and colonization from i.v. grafts. It is important to note that although our studies implicate 27HC as a mediator of the pro-metastatic actions of cholesterol they do not rule out the possibility that other metabolites of 27HC, such as cholestenoic acid, may also be involved. Furthermore, we have not investigated how 27HC treatment might also alter other metabolites, in particular cholesterol-5,6-epoxides. These epoxides have been shown have anti-tumor properties, and in fact tamoxifen is a ligand for the enzyme responsible for their synthesis (cholesterol epoxide hydrolase, ChEH). Treatment of breast cancer cells with tamoxifen leads to the accumulation cholesterol-5,6α-epoxide, contributing to the efficacy of this drug^37^. Furthermore, cholesterol-5,6α-epoxide can react with histamine to form dendrogenin A, a metabolite with tumor-suppressing activity^24^. Therefore, it will be important for future studies to consider the complete cholesterol-homeostatic landscape.

We and others have shown previously that 27HC can function as an endogenous SERM^38, 39^. In models of the cardiovascular system, for instance, 27HC behaves as an ER-antagonist, while it functions as a partial agonist in osteoblasts and in ER-positive breast cancer cells^39–42^. In animal models of ER-positive breast cancer, the partial agonist activity of 27HC is sufficient to support tumor growth^16, 17^. It has also been demonstrated that 27HC can function as a robust LXR agonist and that similar to other synthetic LXR agonists it induces the expression of markers of the epithelial to mesenchymal transition in breast cancer cells (EMT)^16, 40, 43^. However, induction of this program by pretreating cells with 27HC prior to their engraftment in mice, resulted in a very modest increase in metastasis as compared to that which was observed when cells were engrafted into 27HC pretreated mice (ref. ^16^ and Fig. 2a). Thus, although 27HC can induce the expression of some of the key markers of EMT, it is likely that the cancer cell extrinsic actions of 27HC are most important in facilitating metastasis. Indeed, the pro-metastatic activities of 27HC were readily apparent in studies designed to isolate the activities of this oxysterol to the host. Although subtle, an increased metastatic burden was observed in mice grafted i.v. with cancer cells and subsequently treated with 27HC in a chronic manner. We believe that this may represent the promotion of ‘‘secondary’’ seeding events. This observation is significant as it suggests that inhibition of 27HC synthesis or action may be beneficial in patients who have already been diagnosed with metastatic disease.

In this study, it was demonstrated that the pro-metastatic effects of 27HC were entirely dependent on the presence of clodronate-liposome sensitive myeloid cells, which are known to contribute significantly to the metastatic process^27, 28, 44–46^. The role of granulocytes in metastasis is somewhat enigmatic in that they have been shown to have both positive and negative effects on tumor biology, these effects being elaborated in a context specific manner. However, neutrophil infiltration at the distal site has been shown to be required for metastasis and such infiltration has been attributed to activation of CXCR2 and resulting downstream signaling events^33, 34, 47^.

Immune cell profiling indicated both IMs and PMNs were increased in the metastatic lungs of mice treated with 27HC. Since the motility of PMNs is induced by CXCR2 signaling, and considering previous reports that oxysterols can activate CXCR2, we tested the possibility that the effect of 27HC on metastasis resulted from its ability to activate CXCR2 in PMNs. Two important findings in this study support this hypothesis: (1) a small molecule inhibitor of CXCR2 was able to ablate the colonizing effects of 27HC, and (2) 27HC had no effect on cancer cell colonization in animals in which PMNs were immune-depleted. On the other hand, while depletion of IMs reduced basal colonization, 27HC continued to promote cancer cell colonization under these conditions.

T-cell subtypes have been shown to have dramatic effects that promote or inhibit cancer progression. Specifically, CD4^+^ CD25^+^ T-regulatory cells (or Tregs) have been shown to suppress anti-tumor immune responses by CD8^+^ T cells^48^. Further, CD8^+^CD3^+^ cytotoxic T cells naturally respond to the presence of cancer cells and initiate apoptotic mechanisms in tumor cells, but their effectiveness is neutralized by host factors produced by cells within the tumor environment^49, 50^. Bridging the gap between these axes are γδ T-cell receptor (TCR)^+^ CD3^+^ T cells; a rare population of T lymphocytes whose characteristics straddle those of innate and adaptive immunity. These lymphocytes are thought to develop from the same late precursor as αβ TCR^+^ T cells, but gain their specific TCRs during development in the thymus^51^. In contrast to αβ TCR^+^ T lymphocytes, γδ-T cells can be activated through direct recognition of ligands, and can initiate rapid immune responses. Though earlier reports have indicated their anti-cancer activity^52–54^, recent reports provide compelling arguments for their cancer-promoting role. It has been shown that γδ-T cells infiltrate tumors, and encourage metastasis by secreting IL-17, which modulates the polarization of neutrophils and that this results in the suppression of cytotoxic CD8^+^ T-cell proliferation and effector phenotype^33^. Our work suggests a feed-forward mechanism whereby 27HC stimulates the recruitment of PMNs which are required for the proliferative action of 27HC on γδ-T cells, and based on previous reports^33^, the γδ-T cells would then promote further PMN recruitment in addition to polarizing PMNs into an immune-suppressive subtype. Although we have not specifically investigated PMN polarization, we did find that the induction of certain genes associated with metastasis-associated PMNs by 27HC required the presence of γδ-T cells. The numbers of γδ-T cells are significantly elevated in the tumors of patients with advanced disease, and elevated numbers are a marker for poor prognosis^55^. Most relevant to this study, is the ability of γδ-T cells to recognize and respond to lipid antigens^56^, cancer cell-derived antigens^57^, and mevalonate metabolites in tumor cells^58^. Our results demonstrating the effect of 27HC on this T-cell population supports a model whereby 27HC leads to the activation/recruitment of γδ-T cells and PMNs, the activity of both cell types being ultimately responsible for the suppression of CD8^+^ cytotoxic T cells and pro-colonizing properties of 27HC. The precise molecular mechanisms by which 27HC impacts immune biology in these cells remains to be determined.

Here, we show that 27HC is a major biochemical mediator of the metastatic effects of hypercholesterolemia. Indeed, 27HC is a circulating metabolite that dramatically increases breast cancer metastasis through its interaction with γδ-T cells and PMNs. Although the precise signaling events involved are not yet known, 27HC has previously been shown to interact with the ERs, LXRs, and CXCR2. Of note, a recent report indicates that ER signaling can lead to altered myeloid cell infiltration and activity within the ovarian tumor microenvironment^29^. In addition, a recent report indicates that cholesterol is a ligand for the estrogen-related receptor alpha (ERRα), and modeling would suggest that 27HC might also interact with this receptor^59^. All four of these potential targets are highly amenable to therapeutic intervention. Notable was the observation that genetic or small molecule dependent inhibition of CYP27A1 significantly reduced breast cancer colonization of the lungs in two clinically relevant animal models. While statin therapy may be effective in the short term, a recent ‘‘window of opportunity’’ study indicated that HMG-CoA reductase was upregulated in tumors from patients treated with statins, indicating that downstream targeting (such as CYP27A1 or the effectors of 27HC) will likely be more efficacious^60^. Furthermore, since the effects of 27HC are on the host environment, its effects are likely to be broadly applicable to all solid tumors, and we present evidence that this is the case for colorectal, pancreatic, melanoma, and lung cancers.

## Methods

### Reagents

27HC and GW273297X were synthesized by Sai Life (Hyderabad, India). HFD and its corresponding control (5TJS and 5TJN) were purchased from TestDiet. HCD (2% cholesterol and 0.5% sodium cholate) and its respective CD (0.5% sodium cholate) were purchased from TestDiet (5CNY and 5BRW). 27HC and GW273297X for in vivo administration were solubilized in 40% (2-hydroxypropyl)-β-cyclodextrin at room temperature, sterile filtered (0.2 µm) and then stored at 4 °C prior to subcutaneous injections (20 mg/kg for 27HC, 100 mg/kg for GW273297X). Auto-oxidation of these compounds was not specifically controlled for in their formulation. Antibodies for flow cytometry were purchased from BD Biosciences or R&D systems and used at a working dilution of 1:100 in FACS buffer (2% fetal bovine serum (FBS) in PBS, supplemented with penicillin/streptomycin). Catalogue numbers as follows: CD11b: 557686, CD11c: 563735, Ly6C: 553104, Ly6G: 551461, CCR2: FAB5538A, CD3: 555275, CD4: 553729, CD8: 557682, TCRγ/δ: 563532. Immune depletion antibodies and their isotype controls are as follows: α-Ly6G clone 1A8 from BioLegend catalogue number 400544; α -Ly6C clone HK1.4 from BioXCell catalogue number BE0284; α-TCRγ/δ clone UC7-13D5 and isotype control from BioXCell catalogue numbers UC7-13D5 and BE0091; α-CD8 clone YTS 169.4 from BioXCell catalogue number BP0117; isotype control for α-Ly6G, α -Ly6C and α-CD8 were from BioLegend catalogue number 127632).

### Cell culture

E0771 cells were obtained from Mark Dewhirst (Duke University) and grown in RPMI supplemented with 8% FBS, non-essential amino acids, sodium pyruvate, and penicillin/streptomycin. Met1 cells were obtained from Alexander Borowsky (University of California at Davis) and grown in Dulbecco's Modified Eagle Medium (DMEM) with the same supplements. All cell lines were routinely tested for mycoplasma. Met1 and E0771 cells were stably transfected with infrared fluorescent protein (iRFP) as previously described^16^.

### Histology

Metastatic lungs were fixed in 10% formalin followed by 70% ethanol, embedded in paraffin and sectioned. Resulting sections were stained with hematoxylin and eosin (H&E).

### FACS quantification of cell types

Metastatic lungs were digested in a collagenase solution in DMEM (1 mg/ml). Resulting dispersed cells were stained with antibodies and analyzed by flow cytometry. Cell populations were identified by flow cytometry based on previous description^32^ as follows: IMs: CD11b^high^; Ly6C^high^; Ly6G^low-negative^; CCR2^+^, PMNs: CD11b^+^; Ly6C^low-negative^; Ly6G^high^, γδ-T cells: CD3^+^; CD4^−^; CD8^−^; γδ-TCR^+^, cytotoxic T cells: CD3^+^; CD4^−^; CD8^+^. Antibodies were used at a working dilution of 1:100.

### Animal studies

All protocols involving animals were previously approved by the Institutional Animal Care and Use Committees of either Duke University School of Medicine or the University of Illinois at Urbana-Champaign. Sample size was determined by previous experience with models, animal availability and end-point statistics (power analysis). C57BL/6 mice were purchased from Charles River Laboratories or Jackson Laboratories. Tcrd^tm1/mom^/J (TCRD^−/−^) mice were purchased from Jackson Laboratories and bred in house. Animals were randomized into indicated dietary regimens or treatment groups prior to graft. Genetic models were randomized post-genotyping, prior to any measurements. Some experiments were blinded from personnel in charge of treatment (as in personnel taking measurements did not know treatment groups) (Figs. 1c, e, g, 2b, and 3b, Supplementary Figs. 3–5, and 7).

Humanized *APOE3* mice were generated previously^22^. They were ovariectomized at ~6 weeks of age and placed on their respective diets for 8 weeks. Daily treatment with indicated ligands commenced 7 days prior to engraftment (i.v.) with E0771-iRFP cells. Atorvastatin was administered by oral gavage (40 mg/kg) and GW273297X by subcutaneous injection (100 mg/kg). Corresponding body weights are presented in Supplementary Fig. 2. Resulting oxysterol concentrations are presented in Supplementary Fig. 4.

MMTV-PyMT^+^;CYP27A1^−/−^ and MMTV-PyMT^+^;CYP27A1^+/+^ mice were previously generated on a mixed FVB/C57BL/6 background as described^16^. Mice were co-housed and placed on their respective diets (CD or HCD) at wean. At ~5 weeks of age mice were ovariectomized by surgical removal of the ovaries under anesthesia. Mice were euthanized when their collective tumor burden reached 2000 mm^3^. RNA was extracted from the lungs and complementary DNA was generated as previously described^16^. Metastasis was assessed using quantitative real-time PCR (QPCR) to quantify PyMT mRNA expression in the lungs^16^. QPCR was performed using a BioRad CFX384 machine and the BioRad iTaq Universal Sybr Green Supermix; PyMT primers are as follows: [GAGTTCTCCAACAGATACACCC] and [TCCCATGGACTCAGACCCGCC]. Corresponding tumor data presented in Supplementary Fig. 3.

Met1 cells were grafted into female FVB mice at least 8 weeks of age. E0771 cells were grafted into ovariectomized C57BL/6 mice at least 8 weeks of age. For tumor studies (Fig. 1a, d), cells were grafted orthotopically into the mammary fat pad, and resulting tumor growth assessed by direct caliper measurement. For colonization experiments, cells were grafted intravenously via the tail vein. For studies involving these cell lines, metastasis was assessed by ex vivo imaging of lungs on a Licor Odyssey (Figs. 1a, b, d, f, 2a, b, 3, and 4c–i), or by counting visible nodules (Figs. 2c and 4j) and normalized to control or placebo as indicated. Briefly, the trachea was cannulated and lungs inflated with a cold PBS solution, removed from the mouse and placed in a cold PBS solution. Visible nodules were counted, or lungs were scanned at an excitation of 700 nm on a Licor Odyssey apparatus.

### Pretreatment model

Mice were pretreated with placebo (40% (2-hydroxypropyl)-β-cyclodextrin), 27HC, GW2973297X or SB225002 daily for 5 days (subcutaneous) at which point treatment was ceased. Twenty-four hours after the final injection, cancer cells were grafted i.v. (tail vein; 500,000 Met1, MC36, B16/F0 or Lewis Lung cells, 10^6^ E0771 or KPC915 cells). Clodronate liposomes or PBS liposomes (VU Medisch Centrum) were injected i.v. (0.2 ml) 24 h and again 4 h prior to cell engraftment. SB 225002 was administered 2×/day at a dose of 1 mg/kg during the 27HC pretreatment period. Immune depletion studies were done by injecting mice during the 27HC pretreatment period with 400 and 100 µg of the antibodies or their respective isotype controls 36 and 12 h (respectively) prior to engraftment with Met1-iRFP cells. Depletion of respective immune populations was confirmed 24 h post-inoculation by FACS analysis. Pretreatment with GW273297X was similar as for 27HC pretreatment experiments. For Fig. 2c, 27HC was co-administered with GW273297X for one group.

For early tumor studies, 8-week-old female C57Bl/6 mice (Charles River, Wilmington, MA) were ovariectomized and allowed to recover for 1 week. E0771 cells were then injected into an axillary mammary fat pad. Two days after E0771 engraftment, mice were treated daily with subcutaneous injections of either 27HC (20 mg/kg) or vehicle (cyclodextrin). Eight days post-engraftment, mice were euthanized and tumors removed for flow cytometric analysis.

### Plasma cholesterol and oxysterol quantification

Plasma was sent to the Duke Proteomics and Metabolomics Shared Resources for analysis by ultraperformance liquid chromatography/electrospray ionization/tandem mass spectrometry using a customized method allowing chromatographic resolution of the isobaric hydroxycholesterol species. A standard curve of 27HC (0, 0.01, 0.1, 0.5, 1, and 5 μM made up in BSA) was generated. Plasma concentrations of 27HC were determined by calculating the ratio of analyte to internal standard within each sample, and using this value as ‘‘*y*’’ in the linear regression equation generated from the standard curve. 24(R/S)-HC, 22(R)-HC, 22(S)-HC, 4b-HC, 7a/b-HC and cholesterol concentrations are semi-quantitative, calculated based on a ratio to each internal standard (stable-isotope dilution).

### In vitro studies of PMNs

Bone marrow-derived cells were isolated by centrifugation on a density gradient containing Histopaque 1077 layered over Histopaque 1119 (Sigma-Aldrich, St Louis, MO). The lower of two resulting bands of cells was collected and plated in complete RPMI media (including 8.85% FBS; Hyclone, Logan, UT). Cells from spleens were resuspended in complete RPMI media, and counted. Bone marrow cells and splenocytes were then co-cultured by seeding a 1:20 ratio of PMN:Splenocytes, and treated with indicated ligands. Cells were incubated for 4 days (Fig. 4a, b) or 24 h (Supplementary Fig. 16) and then harvested for further analysis.

### Migration assays

Cells were seeded in the top of Boyden Chambers with conditioned media from breast cancer cells as well as indicated ligands in the bottom chamber. Staining and quantification were performed as described previously^40^. CXCR2^fl/fl^ mice were originally purchased from Jackson Laboratories. For assays involving PMNs from these mice (Supplementary Fig. 10a), PMNs were infected with lentivirus expressing Cre-recombinase under the control of a CMV promoter (MOI of 1, GeneCopoeia) for 24 h prior to use in the migration assay. Expression of Cre-recombinase and reduction of CXCR2 mRNA was confirmed by QPCR.

### Analysis of TCGA data

TCGA microarray expression data from 817 invasive breast carcinomas was obtained from the C-BioPortal (data set described in refs ^61^^–^^63^). Data from patients whose gene expression for either CYP7B1, CD66b, IL-17, CD3e, or Perforin read ‘‘NaN’’ were discluded leaving 421 usable samples. Array gene expression data was transformed (2^^(Expression^^)^), and parsed based on median CYP7B1 expression. Data (as displayed in Fig. 4l) was tested for normal distribution prior to non-parametric Mann–Whitney test for significance.

### Statistics

Data was assessed for normality and were ln-transformed or a non-parametric test was chosen as appropriate. No replicates were excluded, with the exception of one mouse (Fig. 4c–e) as the cell-graft was not successful. One-way ANOVA followed by a SNK multiple comparison test was used for Figs. 1c, e, g, 2b, c, 3a–e, and 4i–k. Unpaired two-tailed student’s *t*-test was used for Figs. 1a, b, d, f, 2a, d, and 4a–h. Fisher’s Exact test was used for Supplementary Fig. 17. Mann–Whitney tests were used for Fig. 4l. Statistical differences (*p* < 0.05) are indicated on graphs with asterisks and/or lines.

### Data availability

The TCGA data referenced during the study are available in a public repository from the cBioPortal For Cancer Genomics website (http://www.cbioportal.org/). All the data that support the findings of this study are available within the article and its Supplementary Information files or from the corresponding author upon reasonable request.

## Electronic supplementary material

Supplementary Information
