# Supplementary material for: The cholesterol metabolite 27 hydroxycholesterol facilitates breast cancer metastasis through its actions on immune cells
Source: Nat Commun. 2017 Oct 11;8:864. doi: 10.1038/s41467-017-00910-z (PMC5636879; doi:10.1038/s41467-017-00910-z)
Supplement: Supplementary file 1 — Supplementary Information [file 41467_2017_910_MOESM1_ESM.pdf]

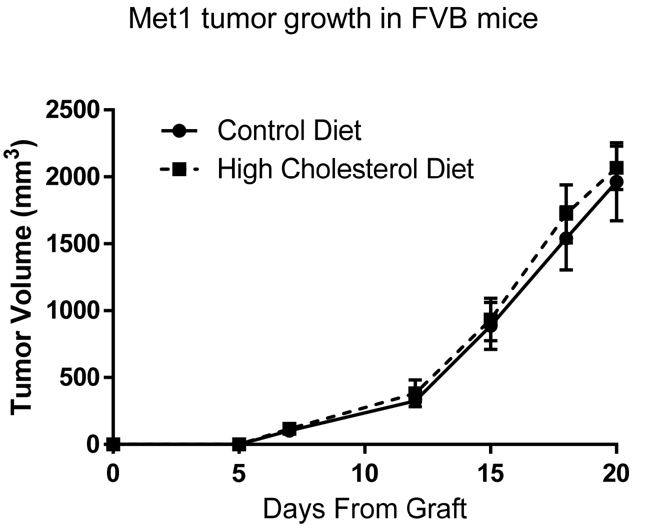

Supplementary Figure 1: A high cholesterol diet does not impact primary tumor growth of murine ER-negative Met1 grafts. Met1 cells were orthotopically grafted into the mammary fat pad of FVB mice on a control or high cholesterol diet, and growth was followed through time by measurement with calipers. Data corresponds to Figure 1a of the main text, where lungs extracted from these mice had significantly higher metastatic tumor burden. Results are depicted as mean +/- SEM, N=10 in each group.

a

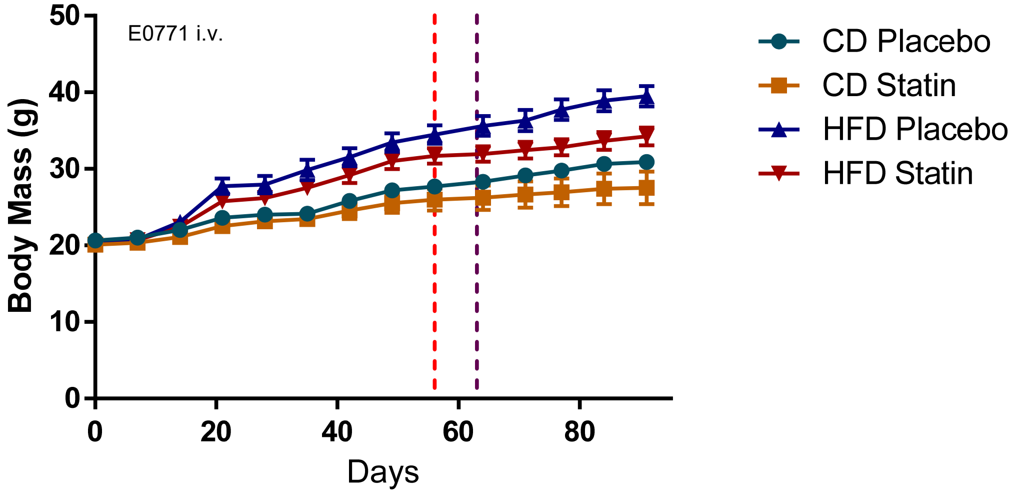

b

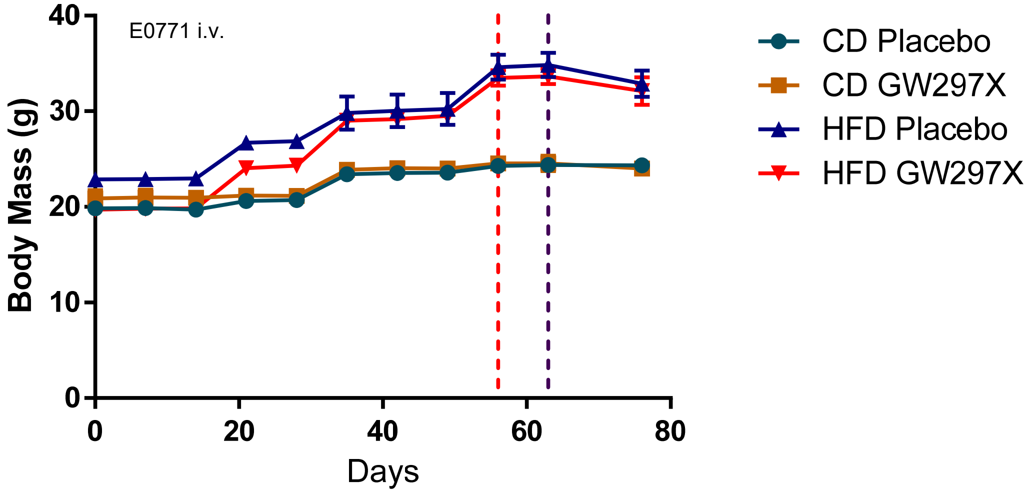

Supplementary Figure 2: (a) Weight change in APOE3 mice on control and high fat diets (CD and HFD respectively). This data corresponds with Fig. 1c. Red dashed line indicates start of statin treatment. Purple dashed line indicates date of E0771 graft (i.v.). Data are presented as mean mass +/-SEM. N=11, 7, 8, 7 for CD Placebo, CD Statin, HFD Placebo, HFD Statin respectively. (b) Weight change in APOE3 mice on control and high fat diets (CD and HFD respectively). This data corresponds with Fig. 1g. Red dashed line indicates start of GW273297X (GW297X) treatment. Purple dashed line indicates date of E0771 graft (i.v.). Data are presented as mean mass +/-SEM. N=5, 3, 7, 6 for CD Placebo, CD GW297X, HFD Placebo, HFD GW297X respectively.

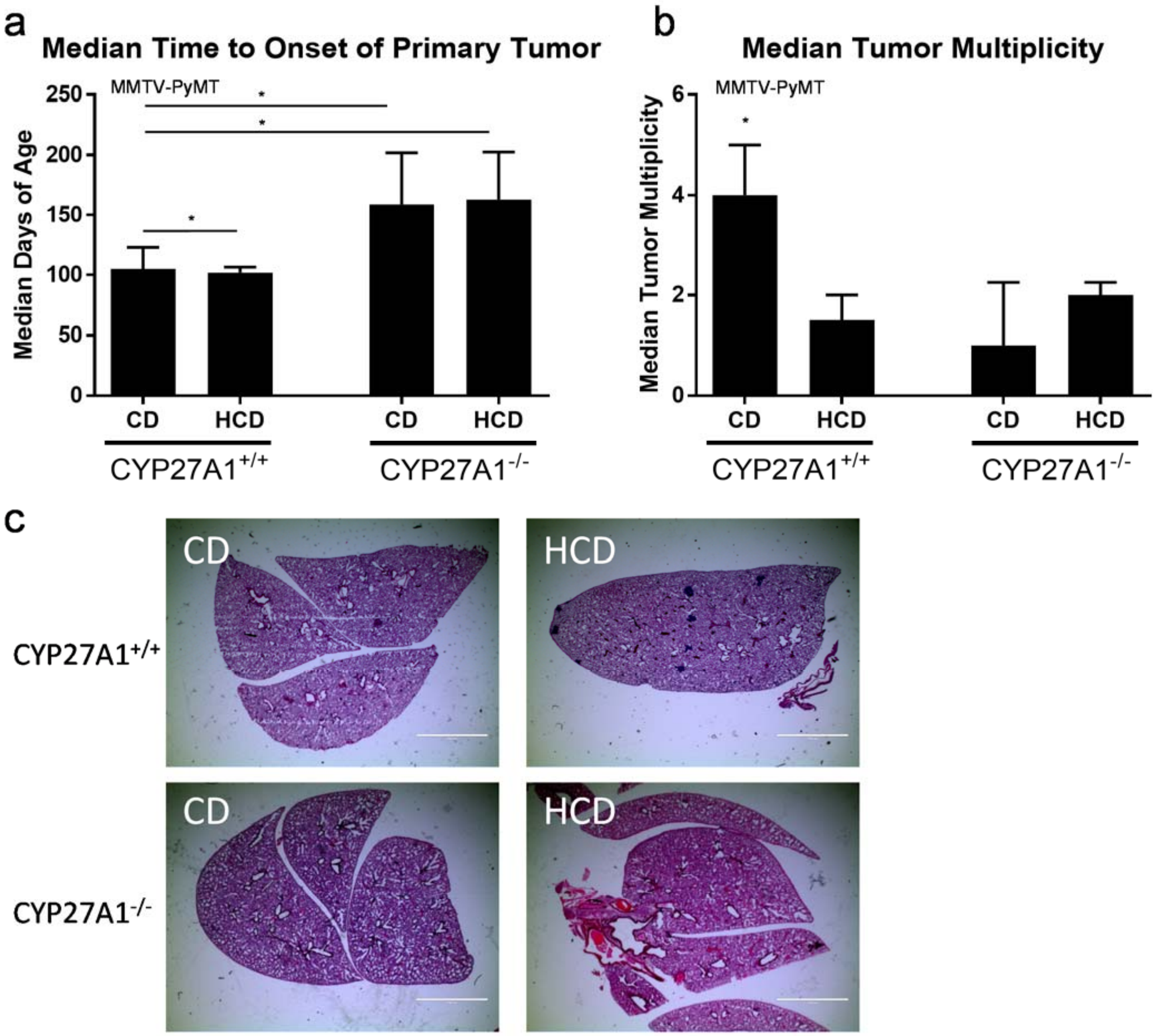

Supplementary Figure 3: (a) Time to primary tumor onset (latency) as determined by palpation, is decreased in MMTV-PyMT mice on a high cholesterol diet (HCD vs. CD), and delayed in CYP27A1$^{-/-}$ mice, regardless of cholesterol intake. Data is displayed as median +/- interquartile range. Asterisks denote statistical significance as determined by Log-Rank (Mantel-Cox) test and reported in Nelson *et al*. *Science*, 2013. N=25, 12, 23, 10 for CYP27A1$^{+/+}$ CD, CYP27A1$^{+/+}$ HCD, CYP27A1$^{-/-}$ CD, CYP27A1$^{-/-}$ HCD respectively. (b) Tumor multiplicity as determined by number of palpable tumors at the time of euthanasia. Data is displayed as median +/- interquartile range. Asterisks denote statistical significance as determined by 1-way ANOVA followed by the SNK test ($p<0.05$). N=22, 12, 22, 10 for CYP27A1$^{+/+}$ CD, CYP27A1$^{+/+}$ HCD, CYP27A1$^{-/-}$ CD, CYP27A1$^{-/-}$ HCD respectively. (c) Representative lung sections from MMTV-PyMT mice indicating that a HCD increases lung metastasis in wildtype mice but not in mice lacking the enzyme responsible for the conversion of cholesterol to 27HC (CYP27A1). Scale bar indicates 2mm. This figure supports data presented in Fig. 1e.

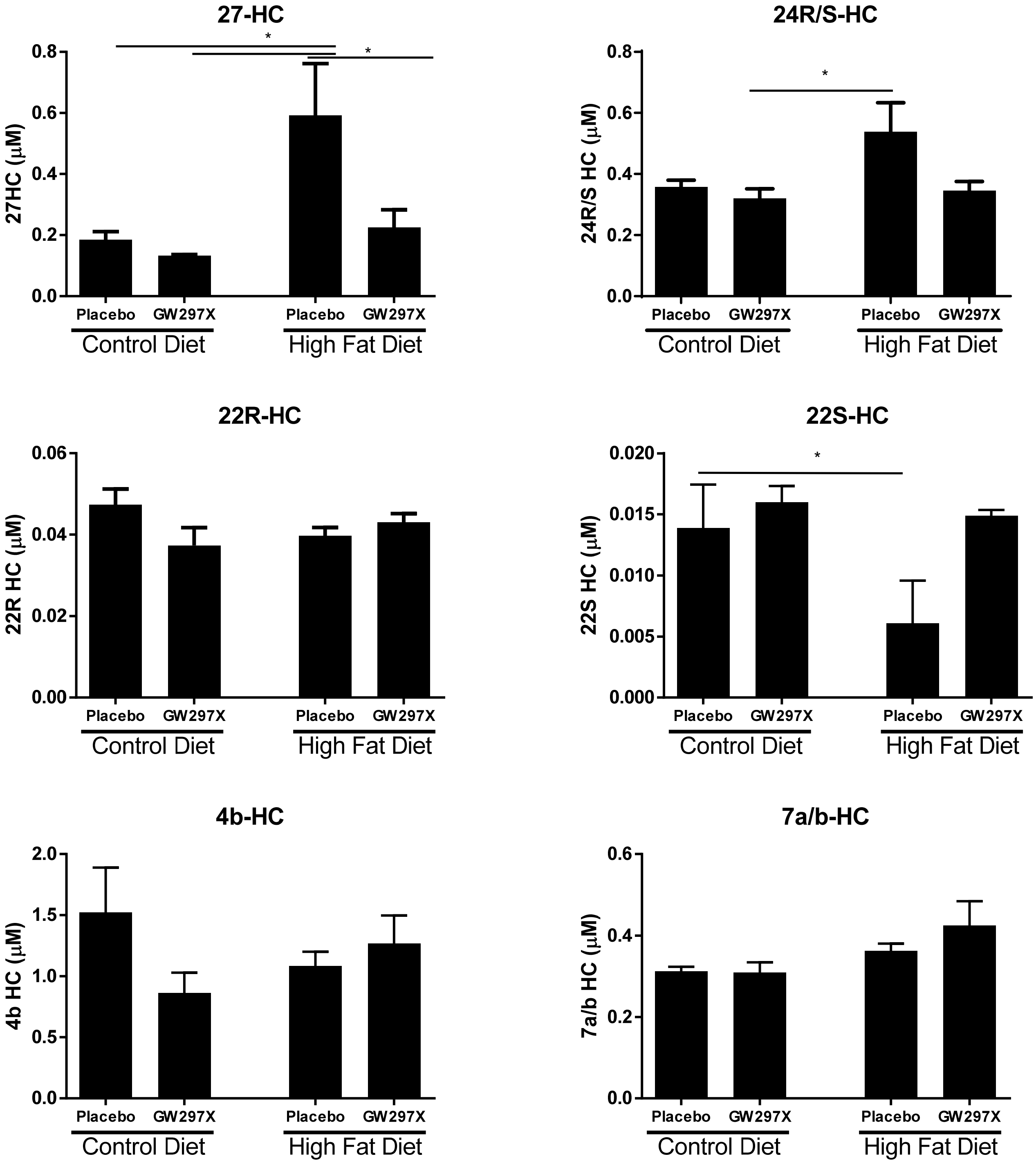

Supplementary Figure 4: Plasma 27HC concentrations are reduced by the CYP27A1 inhibitor, GW273297X, in *APOE3* mice on a high fat diet. Concentrations of other oxysterols were also quantified. Plasma was taken at necropsy and analyzed by LC-MS/MS by the Duke Proteomics and Metabolomics Shared Resources Core. Data corresponds to Figure 1g of the main text. Results are depicted as mean +/- SEM. Asterisks denote statistical significance (P<0.05, One-Way ANOVA followed by a Student Newman-Keuls multiple comparison test). N=5, 4, 4, 4 for Placebo CD, GW297X CD, Placebo HFD, GW297X HFD respectively. Note that two samples in the High Fat Diet placebo treated group had undetectable 22S-HC concentrations and thus were assigned a value of 0μM for this analysis.

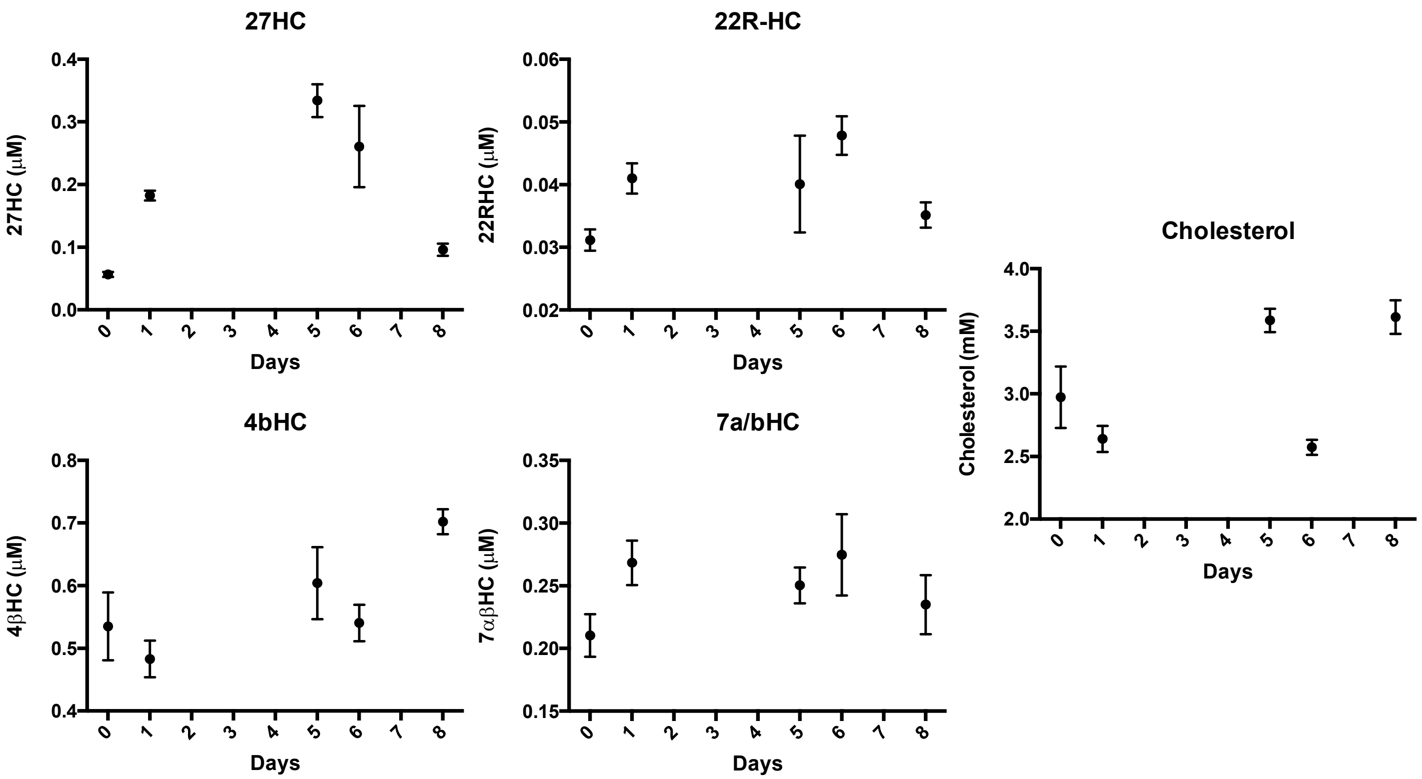

Supplementary Figure 5: Pharmacokinetics of exogenous 27HC administration. C57Bl/6 mice were treated subcutaneously with 20mg/kg 27HC for 1, or 5 days. For mice treated for 5 days, plasma was collected 0, 24 and 72hrs after the last injection (days 5, 6 and 8 respectively). Day 0 represents untreated mice. Cholesterol and oxysterol concentrations were determined by LC-MS/MS. Results are depicted as mean +/- SEM. This data corresponds to Fig. 2 of the main text. N=3 for each time point.

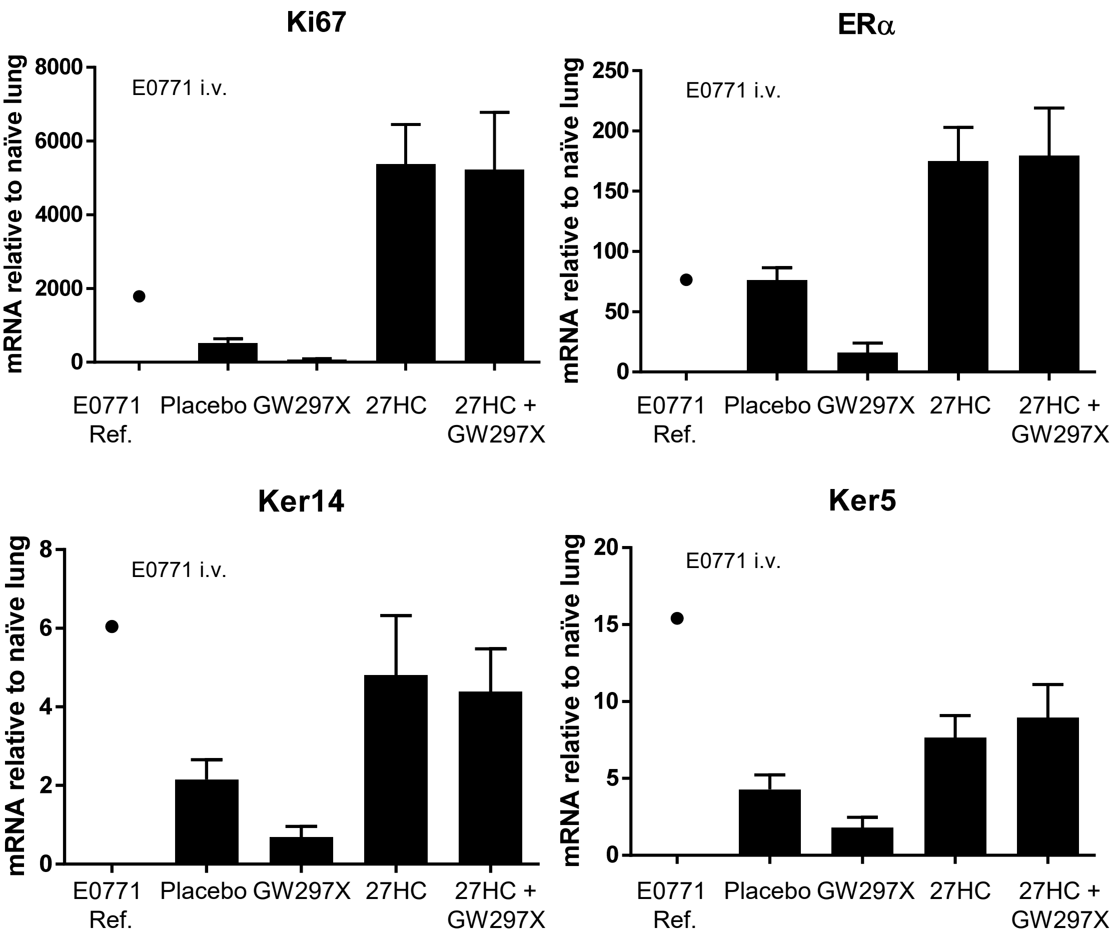

Supplementary Figure 6: Pretreatment with the CYP27A1 inhibitor GW273297X decreases the colonization of the murine lung by E0771 cells, and exogenous 27HC rescues this. Pretreatment model is outlined in Fig. 2a. At necropsy, lungs were harvested and quantitative PCR was used to determine the relative expression of genes which are more abundant in E0771 cells compared to normal lungs. Expression was normalized to lungs from naïve, untreated mice (set at 1). Relative expression in pure E0771 cells is indicated for reference. Results are depicted as mean +/- SEM. Ki67: proliferation marker, ERα: estrogen receptor alpha, Ker14: cytokeratin 14, Ker5: Cytokeratin 5. Results are depicted as mean +/- SEM. N= 5, 5, 5, 4 for Placebo, GW297X, 27HC, and 27HC+GW297X respectively. This data confirms those results in Fig. 2c of the main text where metastatic burden was assessed visually.

a

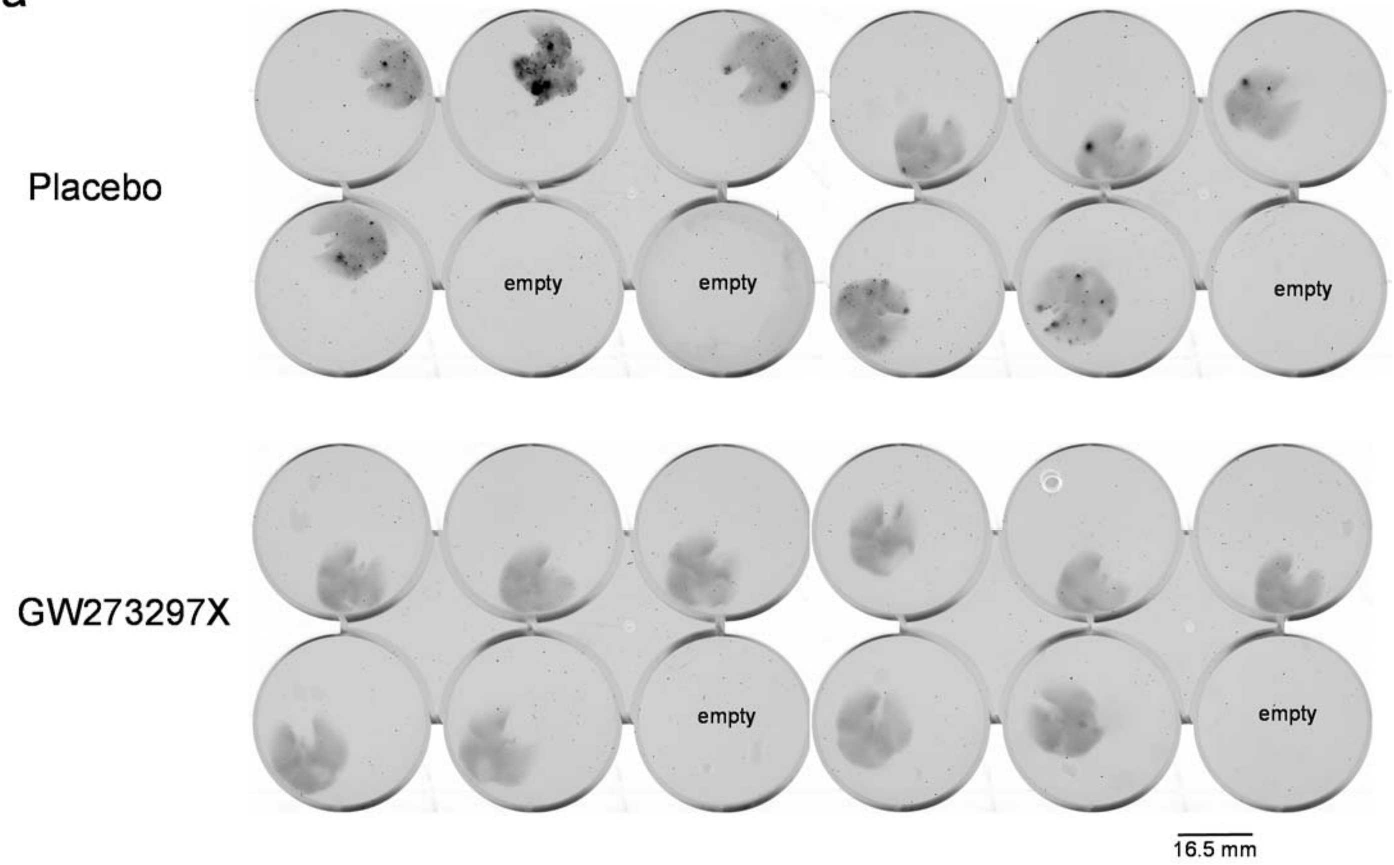

b

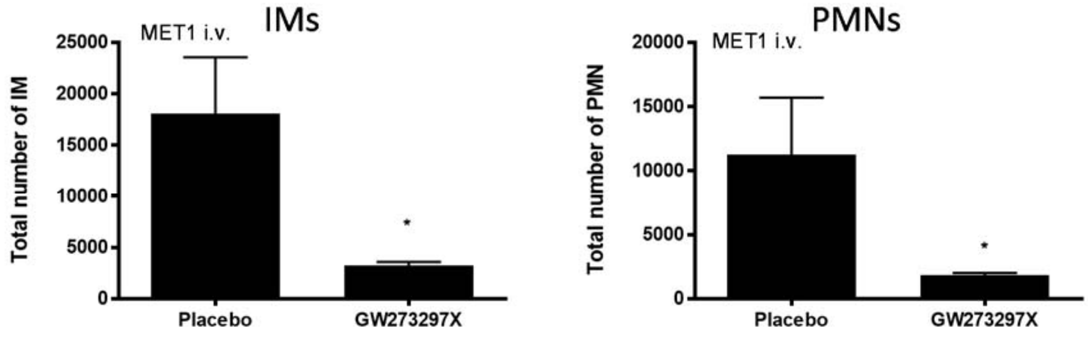

Supplementary Figure 7: Pretreatment with the CYP27A1 inhibitor GW273297X decreases the colonization of the murine lung by Met1 cells. Pretreatment model is outlined in Fig. 2a. Lungs were harvested 5 days post graft. (a) Representative images of iRFP expressing Met1 colonies (depicted as black) within the lungs are presented. Scale bar indicates 16.5 mm. These images correspond to quantified data in Fig. 2d. (b) Flow cytometry was preformed on these lungs to investigate the presence of IMs or PMNs as described in the text. This data corresponds to the experiment outlined in Fig. 2d. Results are depicted as mean +/- SEM. N=9 for Placebo, N=10 for GW27397X. Asterisk denotes statistical significance (P<0.05, unpaired two-tailed student's T-test).

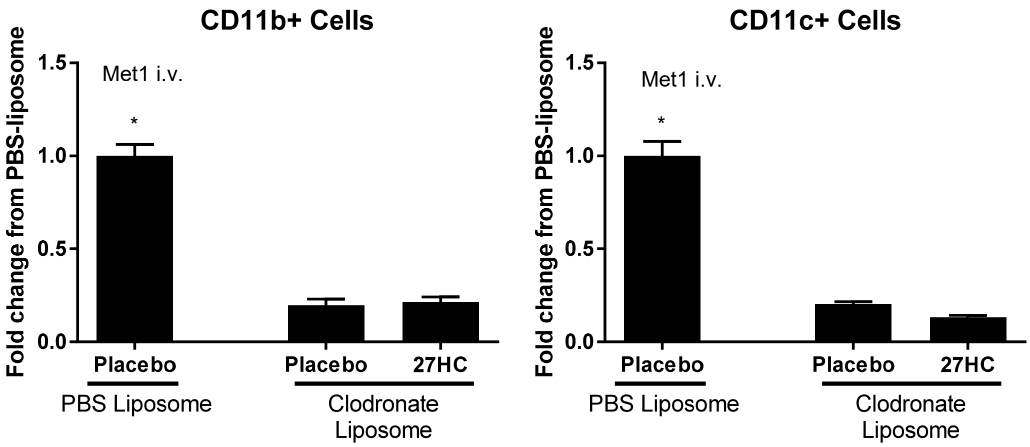

Supplementary Figure 8: Clodronate liposomes effectively reduced CD11b+ and CD11c+ myeloid cells within the lung. For details, see Figure 3a. Results are depicted as mean +/- SEM. N= 4, 5, 5, for PBS Placebo, Clodronate Placebo, Clodronate 27HC respectively. Asterisk denotes statistical significance with other two groups (One-Way ANOVA followed by a Student Newman-Keuls multiple comparison test)

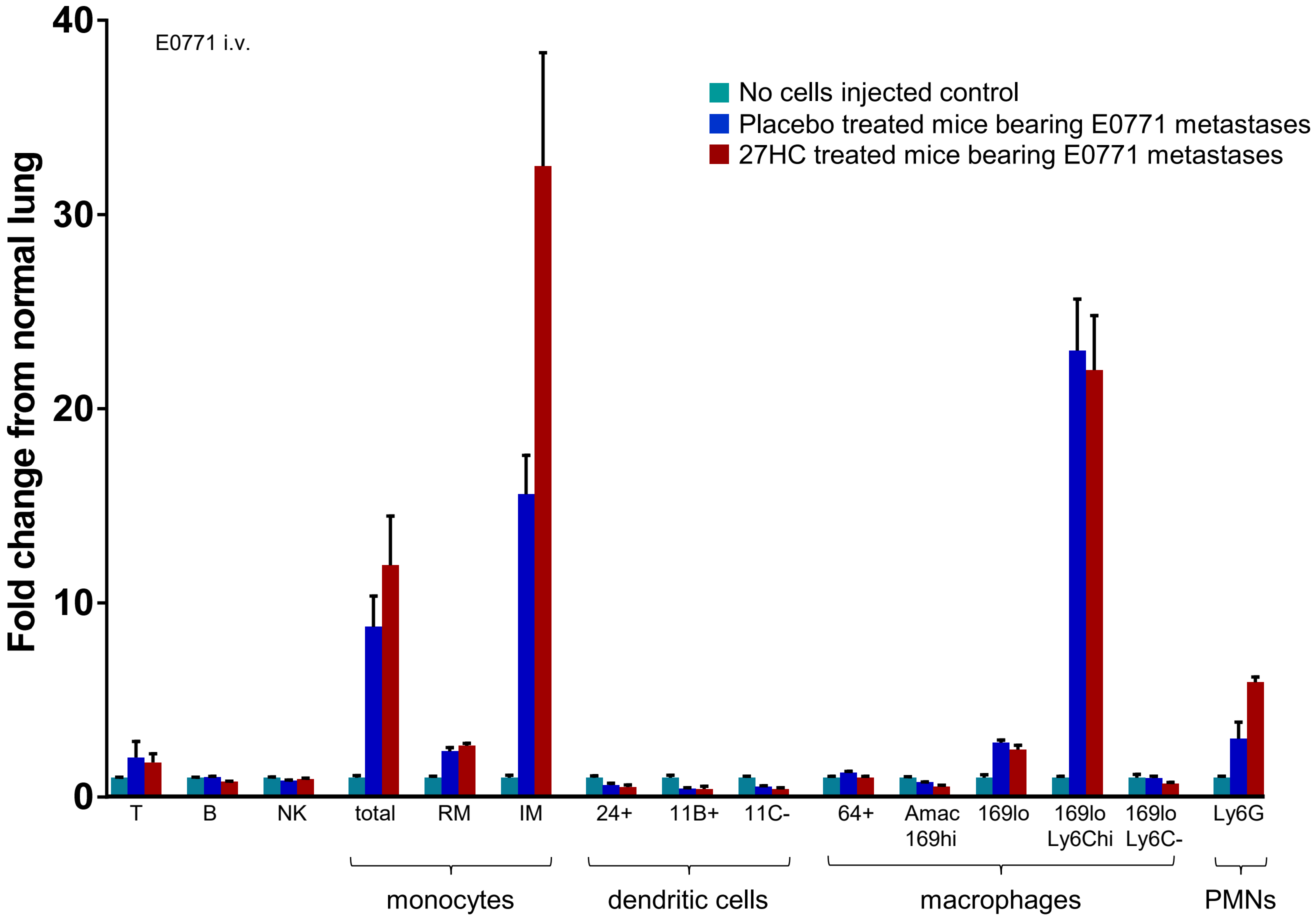

Supplementary Figure 9: Effects of 27HC treatment on leukocyte populations within the lung. Mice were pretreated with either placebo or 27HC and then injected i.v. with either PBS (no cell control), or E0771 cells. Cell types were identified by flow cytometry as described in Yu, et al. (2016) PLoS One 11(3):e0150606. (32). RM: resident monocytes, IM: inflammatory monocytes. 24, 11B, 11C, 64, 169: all refer to cluster differentiation markers (CD). N=5, 3, 3 for No cells, Placebo, and 27HC respectively. Data is presented as mean ± SEM. This data corresponds to Fig. 3b of the main text.

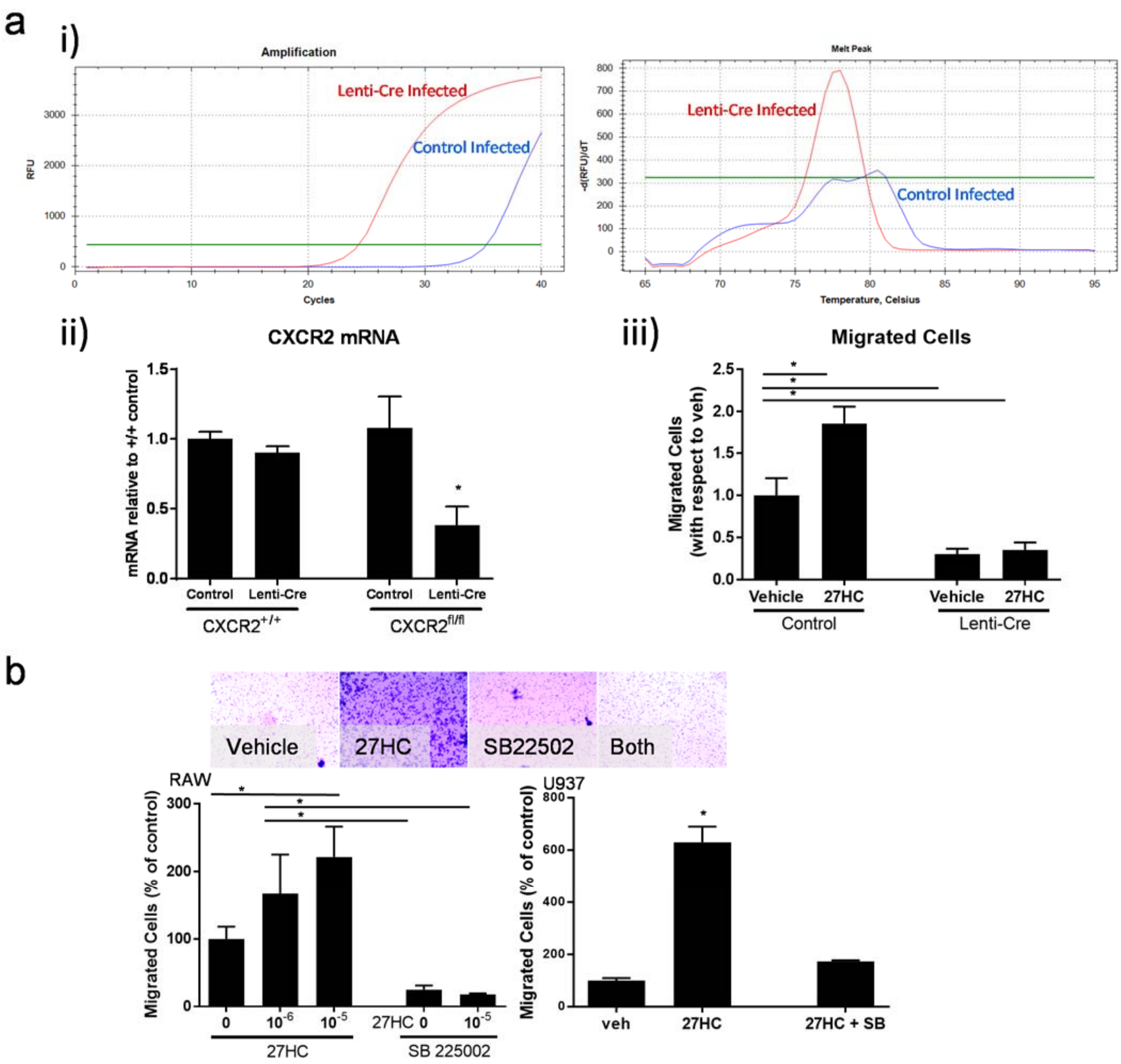

Supplementary Figure 10: 27HC increases migration of PMNs and myeloid cell precursors (RAW 264.7 and U937 cells) in a CXCR2 dependent manner. (a) 27HC induced migration of PMNs is reduced when CXCR2 is removed by the Cre-Lox approach. (i) Representative amplification plot indicating Cre-recombinase mRNA is expressed in PMNs infected with lenti-Cre (red curve, 24hrs post-infection), and corresponding melt curve indicating that the amplification observed for control cells was non-specific. (ii) CXCR2 mRNA was reduced in PMNs isolated from $CXCR2^{fl/fl}$ mice and infected with lentivirus expressing Cre-recombinase, 24hrs post-infection. N=4 in each group. (iii) The increase in migration of PMNs by 27HC is lost in PMNs isolated from $CXCR2^{fl/fl}$ mice and infected with lentivirus expressing Cre-recombinase. N=3 in each group. (b) the small molecule inhibitor of CXCR2, SB225002 attenuates the migratory effects of 27HC in myeloid cell precursor cell lines, RAW264.7 and U937. Representative images of Raw cells migrating through a Boyden chamber membrane are depicted above quantified data. Results are depicted as mean +/- SEM and asterisks denote statistical significance ($P<0.05$, One-Way ANOVA followed by a Student Newman-Keuls multiple comparison test). N=4, 3, 4, 4, 4, from left to right of the RAW panel, and N=3 in each group shown in the U937 panel.

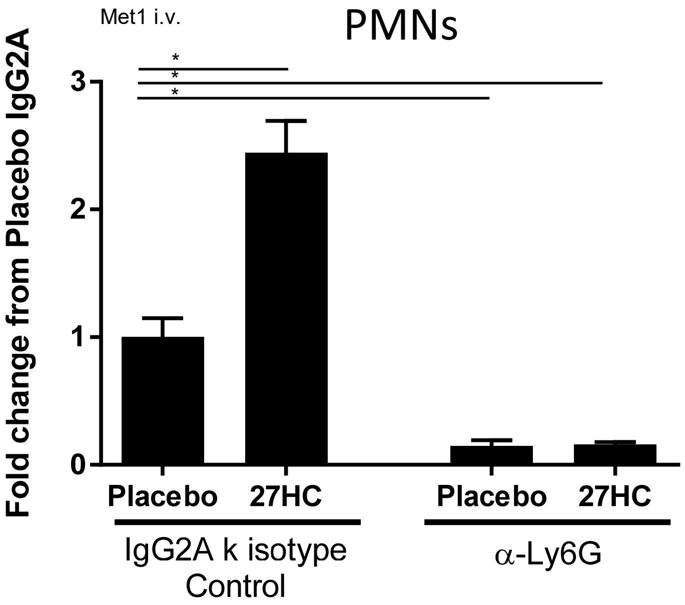

Supplementary Figure 11: Immune depletion strategy effectively decreases presence of Ly6G+ PMNs within the lungs. For details, see Figure 3. Briefly, Mice were pretreated with placebo or 27HC daily for 5 days at which point treatment was ceased. 24hrs after the final injection, Met1 cells were grafted i.v. 36h and 12h prior to engraftment with Met1-iRFP cells, mice were treated with indicated antibodies. 24h after the Met1 graft, lungs were harvested, cells stained and flow cytometry performed. Results are depicted as mean +/- SEM, and asterisks denote statistical significance ($P<0.05$, One-Way ANOVA followed by a Student Newman-Keuls multiple comparison test). N=8, 8, 4, 6 for Placebo isotype, 27HC isotype, Placebo α-Ly6g, 27HC α-Ly6g respectively.

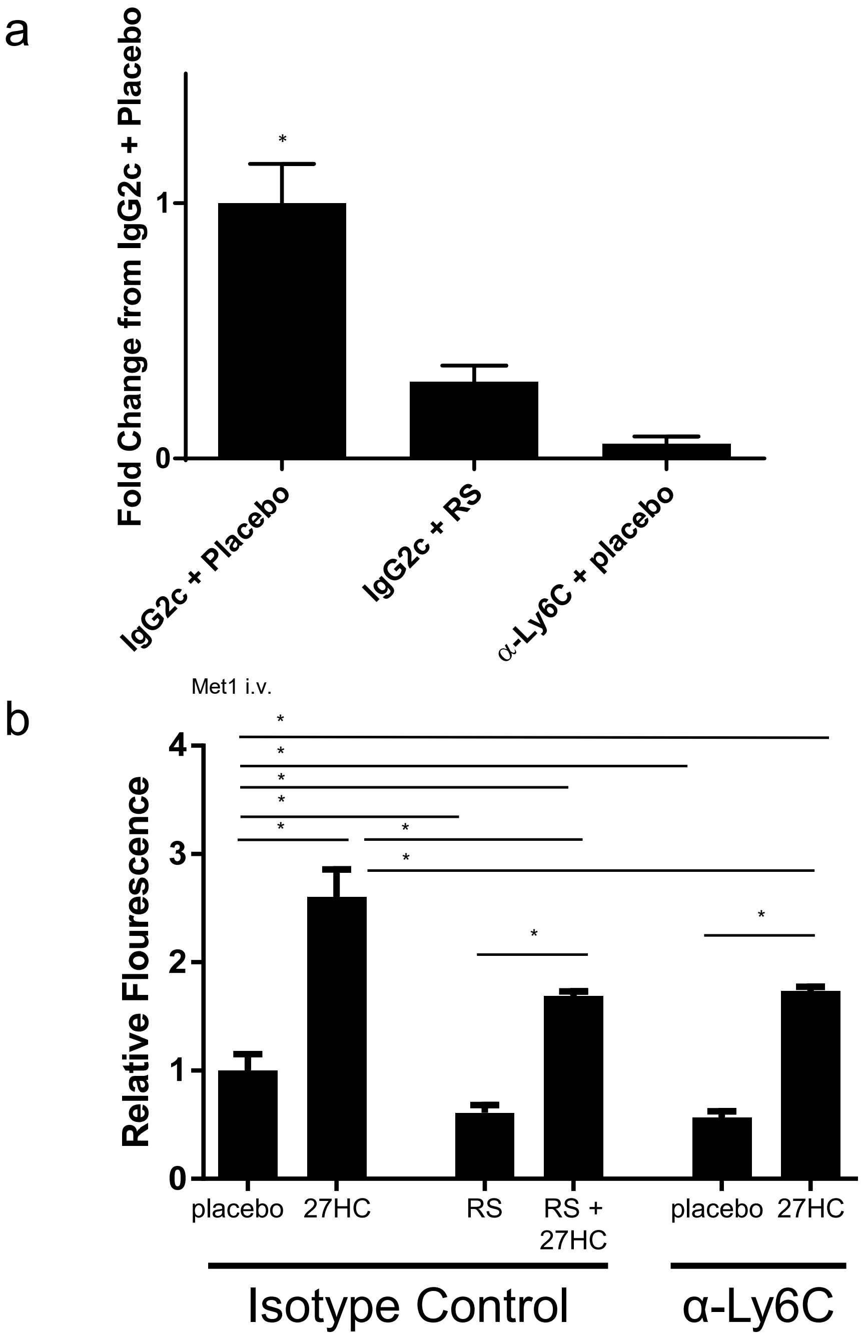

Supplementary Figure 12: Inflammatory monocytes are not primary mediators of the pro-metastatic activities of 27-hydroxycholesterol. (a) Pre-treatment with a CCR2 antagonist (RS-504393; RS) or an antibody against Ly6C effectively reduces the presence of Ly6C+ cells within lungs. N=7, 5, 3 for IgG2c Placebo, IgG2c RS, and anti-Ly6C Placebo, respectively. (b) Pre-treatment of mice with a CCR2 antagonist (RS-504393; RS) or an antibody against Ly6C resulted in reduced metastatic colonization of the lung by Met1 cells. However, 27HC continued to increase metastatic burden. Results are depicted as mean +/- SEM. Asterisks denote statistical significance (P<0.05, One-Way ANOVA followed by a Student Newman-Keuls multiple comparison test). N=6, 6, 6, 5, 6, 6 from left to right. This data corresponds to Fig. 3 of the main text.

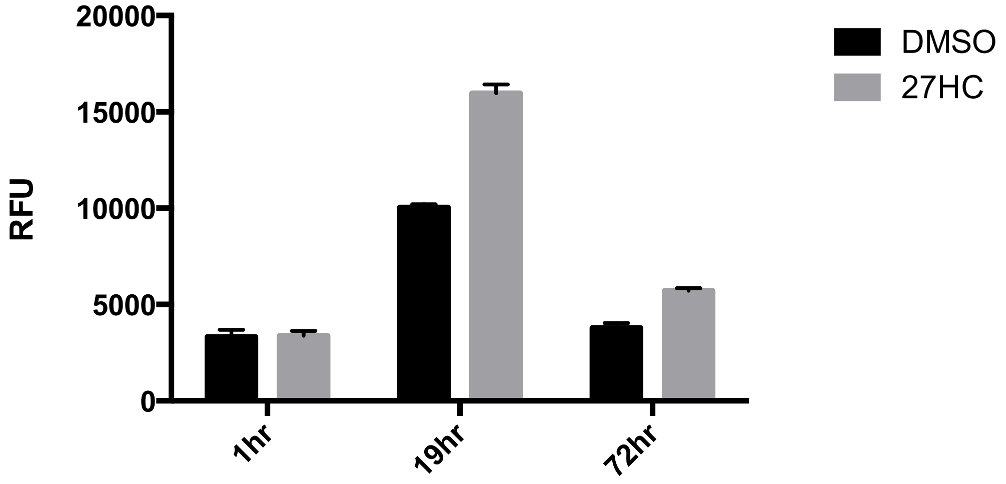

Supplementary Figure 13: PMNs enriched from bone marrow maintain good viability in culture. Viability was assessed by the standard MTT assay (CellTiter-Blue®; Promega) 1hr after isolation, 19hrs and 72hrs after isolation. Y-axis is RFUs subtracting the blank (media alone). Results are depicted as mean +/- SEM. This data corresponds to Fig. 4 of the main text. N=3 in each group.

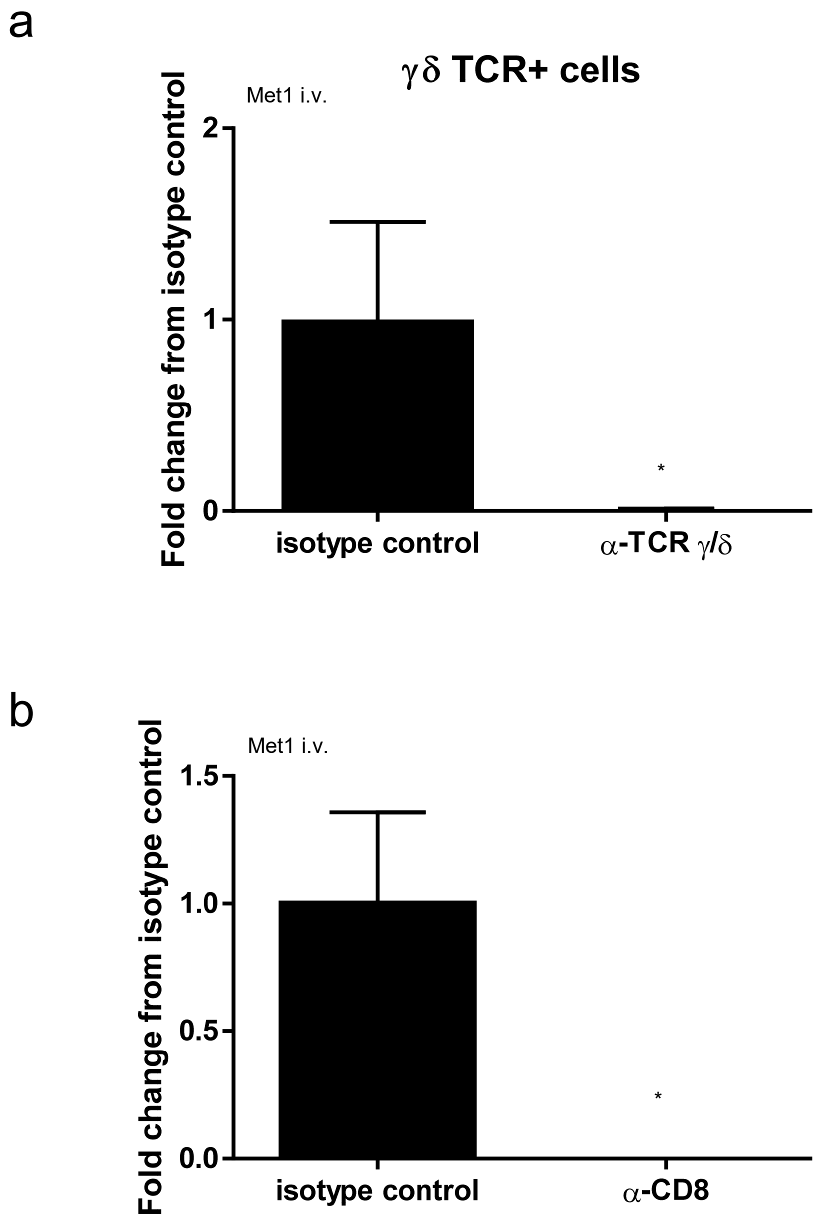

Supplementary Figure 14: Immune depletion strategy effectively decreases presence of (a) γδT-cells or (b) $CD8^+$ T-cells within the lungs. For details, see Figure 4 of the main text. Results are depicted as mean +/- SEM, and asterisks denote statistical significance ($P<0.05$, Unpaired two-tailed student's T-test) . N=3 in each group.

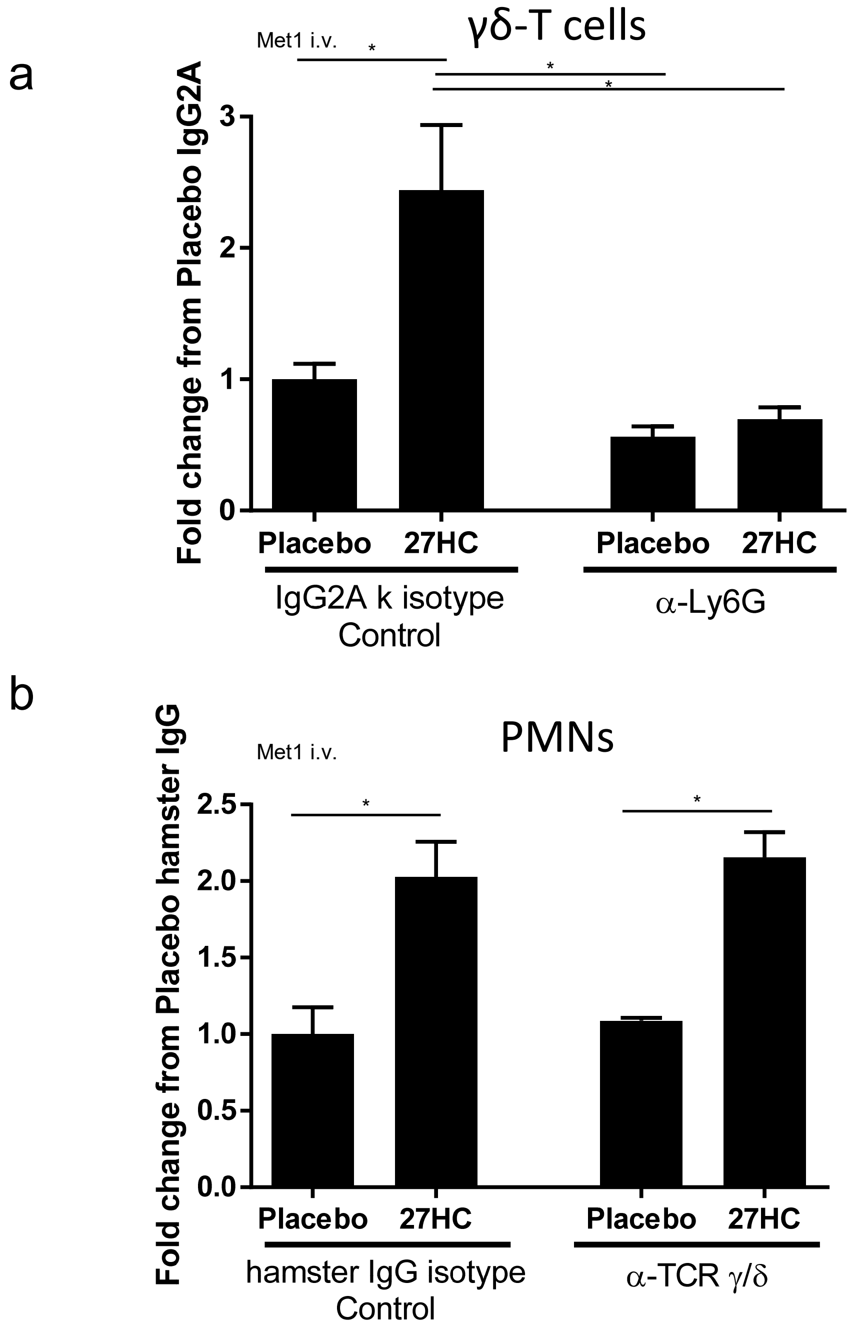

Supplementary Figure 15: (a) 27HC increase in γδ-T cells is absent in mice that have been immune depleted of Ly6G+ PMNs. (b) On the other hand, 27HC continues to increase PMNs in mice that have been immune depleted of γδ-T cells. For details see Figs. 3 and 4. Briefly, Mice were pretreated with placebo or 27HC daily for 5 days at which point treatment was ceased. 24hrs after the final injection, Met1 cells were grafted i.v. 36h and 12h prior to engraftment with Met1-iRFP cells, mice were treated with indicated antibodies. 24h after the Met1 graft, lungs were harvested, cells stained and flow cytometry performed. Results are depicted as mean +/- SEM, and asterisks denote statistical significance ($P<0.05$, One-Way ANOVA followed by a Student Newman-Keuls multiple comparison test). (a) N=8, 8, 4, 6 for Placebo isotype, 27HC isotype, Placebo α-Ly6g, 27HC α-Ly6g. (b) N= 3 each.

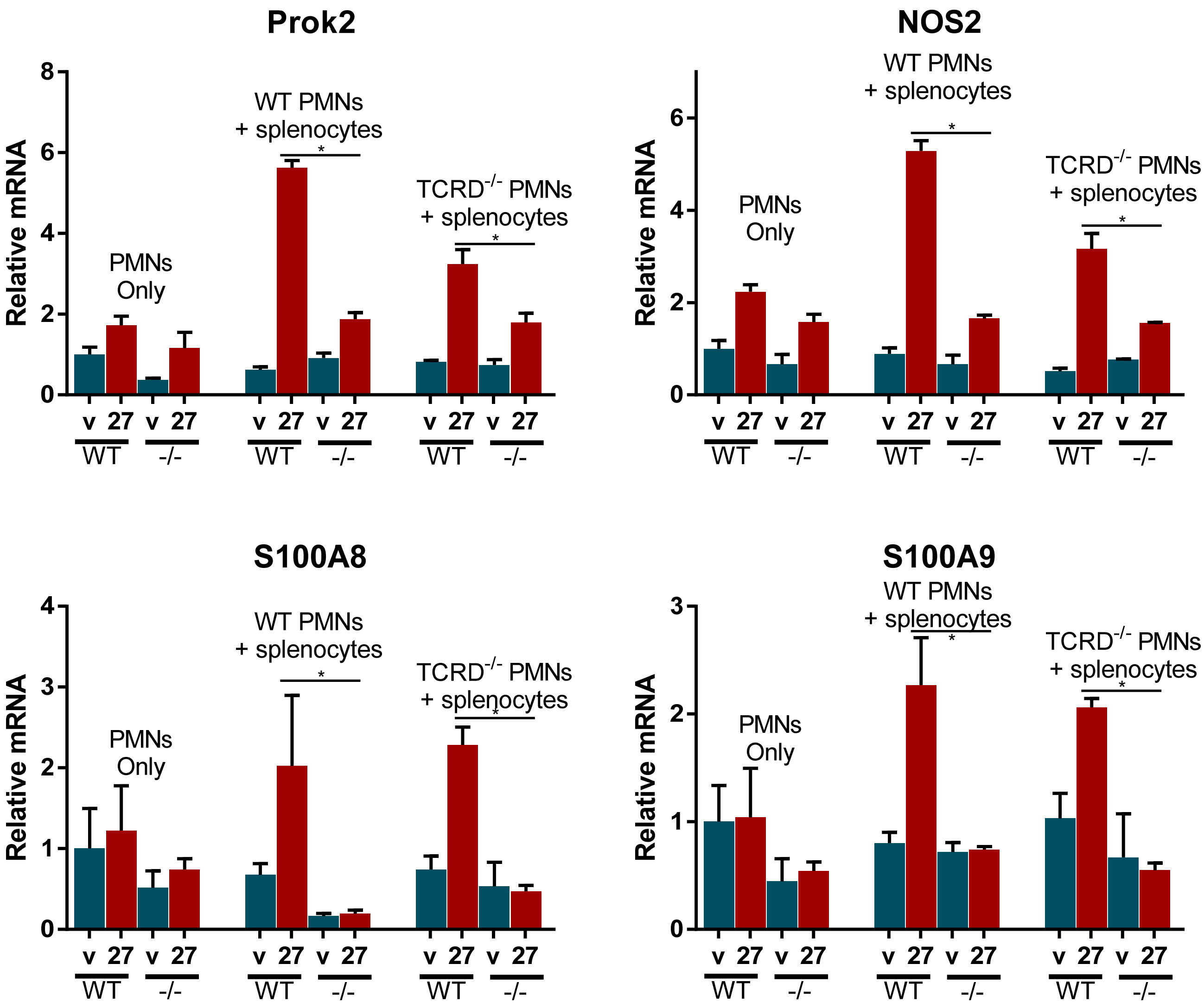

Supplementary Figure 16: 27HC increases the expression of genes associated with tumor associated PMNs, the full induction of which likely requires γδ-T cells. Bone marrow derived PMNs and splenocytes from wildtype (WT) or TCRD$^{-/-}$ (-/-) mice were cultured alone or together (similar to Fig. 4a-b). Cells were treated with vehicle (DMSO; v) or 27HC (10μM) for 24hrs, at which time mRNA was isolated for quantitative RT-PCR. Gene names are indicated above graphs (Prok2, NOS2, S100A8, S100A9). Data from splenocytes cultured alone are not shown. Cultures were run in duplicate and repeated with three independent wildtype and 3 independent TCRD$^{-/-}$ mice. Duplicates were averaged and used to calculate mean +/- SEM (N=3). Asterisks denote statistical significance between indicated 27HC-treated groups (P<0.05, One-Way ANOVA followed by a Student Newman-Keuls multiple comparison test). For clarity other significant differences between other groups are not indicated.

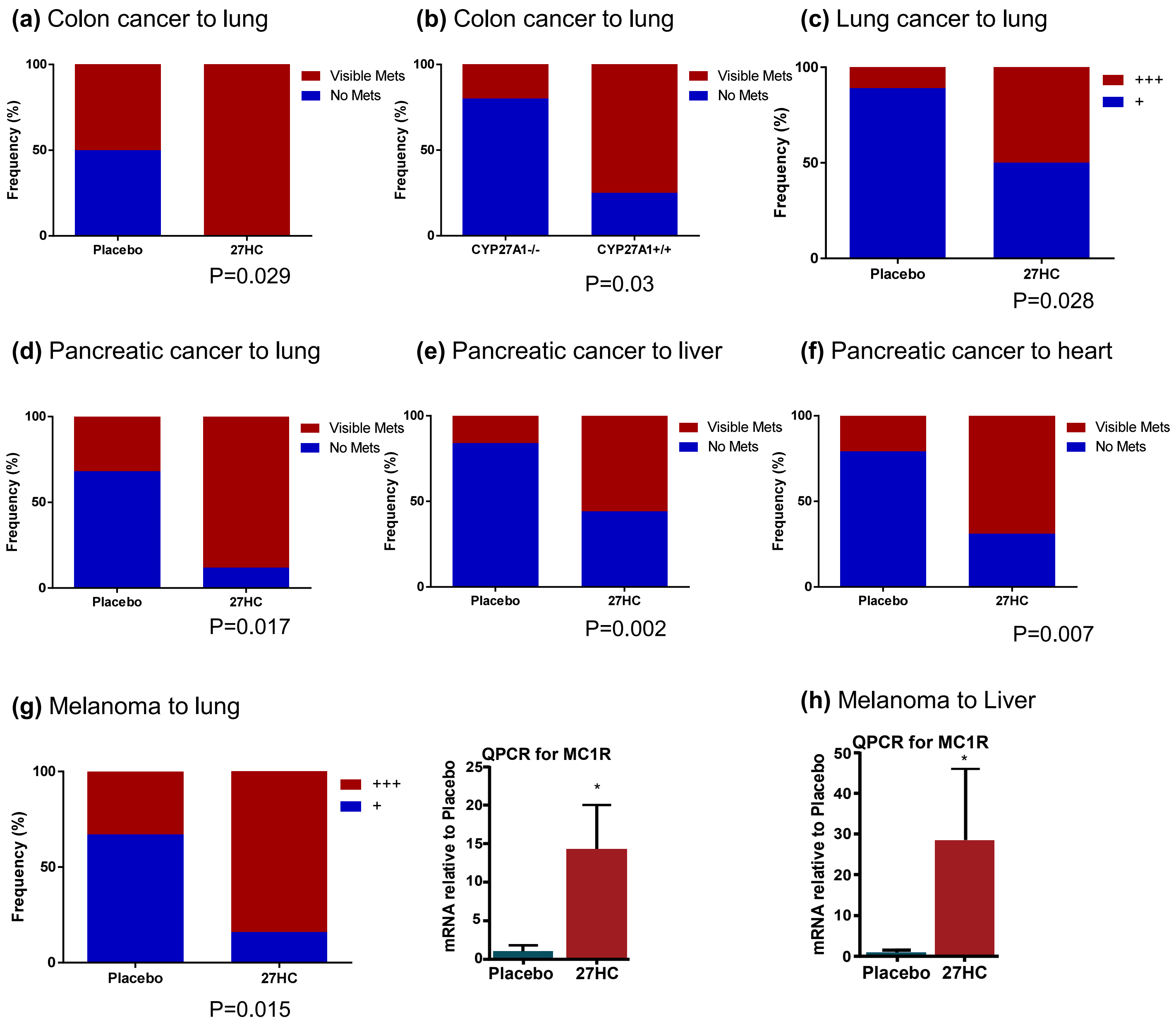

Supplementary Figure 17: 27HC promotes the colonization of cells derived from other solid tumors. With the exception of (b), mice were pretreated with vehicle or 27HC for 5d prior to i.v. graft as outlined in Fig. 2. (a) N=8 for Placebo, N=9 for 27HC. For (b), cells were grafted into either wildtype or CYP27A1 knockout mice. N=10 for CYP27A1$^{-/-}$, N=12 for CYP27A1$^{+/+}$. Colon cancer cells: MC36. (c) Lung cancer cells: Lewis Lung. N=18 in each group. (d-f) Pancreatic cancer cells: KPC915. N=19 for Placebo, 16 for 27HC. g) Melanoma cancer cells: B16-F0. N=18 for Placebo, N=19 for 27HC. N=10 in each group for QPCR analysis. For (c) and (g), lungs were parsed into those with a low metastatic burden (+) and a medium to high metastatic burden (+++). For (g), metastatic melanoma nodules in the lung were assessed visually, and verified by QPCR for the melanocortin 1 receptor (MC1R). For (h) metastatic melanoma in the liver was quantified by QPCR for MC1R. N=10 in each group. P-values for a-g were generated by Fisher's Exact Test. For the QPCR results in (g) and (h), asterisks represent statistical significance ($P<0.05$) as determined by a Student's T test.
